# Community structure and diversity of myxobacteria in soils from Inner Mongolia, China

**DOI:** 10.3389/fmicb.2024.1501573

**Published:** 2025-01-22

**Authors:** Zhihua Wu, Songyuan Li, Xuehan Wang, Huirong Liu

**Affiliations:** ^1^College of Life Sciences, Inner Mongolia Agricultural University, Hohhot, China; ^2^College of Ecology and Environment, Baotou Teachers’ College, Baotou, China; ^3^Jilin Institute for Drug Control, Changchun, China

**Keywords:** myxobacteria, community structure, diversity, species composition, high-throughput sequencing technology, soil physical and chemical properties

## Abstract

Myxobacteria are a special kind of Gram-negative bacteria that can slide and produce a variety of bioactive substances against bacteria, fungi, and viruses. It has great development and research value in medicine and agriculture. Although myxobacteria have become a research hotspot at home and abroad, there are few systematic studies on the relationship between its diversity, geographical location, and environment factors. In order to solve these problems, 133 soil samples were collected from the east to the west of Inner Mongolia Autonomous Region and divided into five groups. The water content, pH, organic matter, available phosphorus, hydrolytic nitrogen, and available potassium content of soil samples were determined by national standards and other methods. The quantitative assessment of the abundance of myxobacteria in the soil sample was performed by quantitative real-time PCR. The composition of myxobacteria in the soil was determined by 16S rRNA high-throughput sequencing technology to analyze the differences in the community structure of myxobacteria among different groups, explore the relationship between the diversity of myxobacteria resources and the distribution and physical and chemical properties of the soil, and predict and analyze its community function. The results showed that there were abundant myxobacteria resources in the soils of Inner Mongolia, and the average relative abundance of myxobacteria in the soil samples from the central part of Inner Mongolia was higher than that in the eastern and western parts, but the richness and diversity of samples from the central to eastern regions were significantly higher than those from the western regions. The myxobacteria resources in the whole region included 10 families and 22 genera, among which the dominant genera were *Labilitrix*, *Sandaracinus*, *Archangium*, and *Haliangium*. The analysis of the species composition of myxobacteria among different groups found that the distribution of soil and soil type had an impact on the species composition of the samples. The species with significant differences in relative abundance among the five groups of samples were *Labilitrix*, *Archangium*, *Sandaracinus*, *Minicystis*, *Polyangium*, and *Myxococcus*. In addition, the latitude and longitude of the sampling point and the soil pH, water content, available phosphorus content and organic matter content had a greater impact on the myxobacteria community structure of samples, while the altitude of the soil sample and the contents of available potassium and alkaline nitrogen had a relatively small impact. Our data suggest that the distribution, type and nutrient composition of soil samples have an impact on the relative abundance and species composition of myxobacteria community. The completion of this work can provide basic data for the in-depth study of myxobacteria in the soil.

## 1 Introduction

Myxobacteria are a group of Gram-negative bacteria with social behavior, complex multicellular behavior, and morphogenesis (Muñoz-Dorado et al., 2016). Myxobacteria have remarkable social characteristics, such as the ability to coordinate feeding (Livingstone et al., 2017), movement (Nan and Zusman, 2011), and formation of fruiting bodies through signal conduction and induction between cells (Ye et al., 2020). Compared with other bacteria, myxobacteria are a kind of advanced prokaryotic group “living on the edge of eukaryotes.” They can produce many bioactive substances (Thiery and Kaimer, 2020) with novel structures and unique mechanism of action (Herrmann et al., 2017). They have become a “micro-factory” of bioactive secondary metabolites (Wenzel and Müller, 2009). The current research on myxobacteria focuses on applied molecular biology, developmental biology, taxonomy, and biotechnology. However, there is still lack of ecological research on the community structure and diversity of soil myxobacteria.

Myxobacteria are widely distributed and are found in soil, desert, and lake water, and sometimes on the surface of plant leaves (Dawid, 2000). In recent years, traces of myxobacteria have also been found in extreme conditions such as deserts (Mohr et al., 2018a) and oceans (Li et al., 2002) with high osmotic pressure, acidity (Mohr et al., 2017), low oxygen, salt, and alkali (Zhang et al., 2013), but they prefer to grow in native soil, or soil rich in microorganisms and organic matter (Liu et al., 2019). Myxobacteria can produce rich and diverse secondary metabolites with novel structures, and have good resistance to bacteria, viruses, fungi, cancer cells, malaria, etc. It has broad application potential in drug research and development, biological pesticides, and ecological management. However, it is difficult to isolate and cultivate myxobacteria, so the true diversity of myxobacteria is still unknown, and the detailed relationship between taxonomic units and different environments and the distribution mode of myxobacteria have not been thoroughly studied, which seriously limits the exploration, development, and utilization of myxobacteria resources.

Inner Mongolia has special geographical environment conditions (Chen and Gong, 2005; Xu et al., 2001). Stretching across northeast and northwest regions of China, Inner Mongolia is the provincial administrative region with the largest longitude in China, with a straight-line distance of more than 2,400 km from east to west and a span of more than 1,700 km from north to south. The amount of precipitation is affected by the terrain and the distance of the ocean, decreasing from 500 to 50 mm from east to west. The corresponding climatic zones are distributed in a zonal pattern, gradually transitioning from humid and semi-humid areas to semi-arid and arid areas from east to west. Vegetation is also distributed regularly in longitude zonality. From east to west, there are forest, grassland, semi-desert, and desert vegetation. The soil in this area is also distributed in an obvious meridional zone, with phaeozem, dark brown soil, chernozem, chestnut soil, brown calcic soil, brown desert soil, and gray desert soil in sequence from east to west. Under these special geographical environmental conditions, there are bound to be rich and diverse myxobacteria resources. However, so far, there have been few reports on the distribution of myxobacteria resources under special environmental conditions in Inner Mongolia.

Therefore, the purpose of this article is to study the community structure and diversity and analyze the community function of myxobacteria in Inner Mongolia by high-throughput sequencing technology, which will provide basic data for the in-depth study of myxobacteria in this area.

## 2 Materials and methods

### 2.1 Soil sample collection

Myxobacteria are naturally and extensively dispersed in the soil ecosystem, but these species remain relatively unexplored among microbes (Thakur et al., 2018). According to the different types of soil, this study collected 133 soil samples from the 5 to 10 cm surface layer of soil in the Inner Mongolia Autonomous Region by using the five-point sampling method. One part was stored at 4°C for the determination of its physical and chemical properties (Wang et al., 2016), and the other was stored at −80°C for DNA extraction and microbial analysis. According to the distribution of soil samples from west to east, the samples were divided into the five groups and the basic information of soil samples are provided in Supplementary Table 1.

### 2.2 Determination of soil parameters

The moisture content of the samples was determined by drying method (Ye and Zhang, 1984). Briefly, the sample was weighed, then dried (usually for a prescribed period of time under specific conditions of temperature) and weighed again. The moisture content was calculated based on the initial and final weights of the sample. The pH value was determined on each of the replicate samples using a pH meter (HI 2221 Calibration Check Ph/ORP Meter, Hanna Instruments, United States) according to the Ecology Common Experimental Research Methods and Techniques (Zhang, 2006). Briefly, 5.0 g fresh soil was mixed with 45 ml deionized water in a 100 ml beaker and then placed in a shaker for 30 min before the pH measurement. NaHCO_3_ leaching – molybdenum antimony and colorimetric method was used to determine the available phosphorus of soil samples (Xing et al., 2011). Briefly, 2.5 g air-dried soil was extracted with 0.5 mol/L NaHCO_3_ (pH 8.5) and detected by spectrophotometry at 880 nm. Soil available potassium was determined by tetraphenylborate nephelometry (Li et al., 1982). Briefly, 5.0 g air-dried soil was extracted with 1.0 mol/L NaNO_3_ and detected by spectrophotometry at 420 nm. The alkaline hydrolysis diffusion method was used to determine the content of hydrolyzable nitrogen (Li, 2010). Briefly, the hydrolyzed nitrogen in the soil was alkaline hydrolyzed in a glass diffusion dish. The ammonia gas separated by diffusion was absorbed by borate-indicator solution, and then the content of hydrolyzed nitrogen was obtained by titrating borate-indicator solution with hydrochloric acid standard solution. And the potassium dichromate-volumetric method was used to determine the content of total organic matter (TOM) in the soils (Ji, 2005). Briefly, under heating conditions, the organic matter of the soil was oxidized with excess K_2_Cr_2_O_7_-H_2_SO_4_, and the excess potassium dichromate was titrated with ammonium ferrous sulfate standard solution. The amount of organic matter was calculated from the amount of potassium dichromate consumed according to the oxidation correction factor, and then multiplied by van Bemmelen factor 1.724 to obtain the organic matter content of the soil.

### 2.3 DNA extraction and quantitative real-time PCR

Genomic DNA was extracted from a fraction of soil sample (0.5 g) using the E.Z.N.A. Soil DNA Kit (Omega, United States) according to the manufacturer’s instructions. DNA extraction was repeated three times for each sample and the resulted DNA were pooled and stored at −20°C.

Total DNA of soil samples was extracted and the integrity of genomic DNA was detected by 1% agarose gel electrophoresis. Quantitative real-time PCR (qPCR) was used to quantify copies of bacterial 16S rRNA genes and myxobacterial 16S rRNA genes using specific primers and temperature profiles. Briefly, the target gene was PCR amplified in a 20 μl reaction mixture containing 10 μl SYBR R Premix Ex Taq Kit (TaKaRa), 0.5 μM of each primer, and 5–10 ng template DNA. Bacterial universal primers 1369F (5′-CGGTGAATACGTTCYCGG-3′) and 1541R (5′-AAGGAGGTGATCCRGCCGCA-3′) were used for PCR amplification. Myxobacteria semi-specific primers W5 (5′-GTAAGACAGAGGGTGCAAACGT-3′) and 802R (5′-ACTACCAGGGTATCTAATCCTG-3′) were used for PCR amplification (Jiang et al., 2010). The amplification reaction of primers 1369F/1541R started with predenaturation at 95°C for 1 min and denaturation at 95°C for 15 s, followed by primer annealing at 59°C for 20 s and primer extension at 72°C for 30 s, 40 cycles. The amplification reaction of primers W5/802R started with predenaturation at 95°C for 1 min and denaturation at 95°C for 15 s, followed by primer annealing at 55°C for 20 s and primer extension at 72°C for 30 s. A total of 40 cycles were performed. The qPCR was performed in triplicate, and the specificity of amplification was confirmed by agarose gel electrophoresis and melting curve analysis. The primers were synthesized by Sangon Biological Engineering Technology and Service Co. (Shanghai, China), and the quantification PCR was performed on a Roche LightCycler 480II platform.

### 2.4 PCR amplification and high-throughput sequencing

Myxobacteria universal primers W5 and 802R were used for PCR amplification (Jiang et al., 2010). Ten microliter reaction system: 10 × Top Taq Buffer 1 μl, dNTPs (2.5 mmol/L) 0.8 μl, upstream and downstream primers (2 μmol/L) 1 μl each, Top Taq (5 U/μl) 0.2 μl, template (10 ng/μl) 1 μl, and the sterile double-distilled water was added to 10 μl. The reaction started with predenaturation at 95°C for 5 min and denaturation at 95°C for 30 s, followed by primer annealing at 55°C for 30 s and primer extension at 72°C for 60 s. A total of 11 cycles were performed (the annealing temperature decreased by 0.5°C for each cycle). Then the reaction continued with denaturation at 95°C for 30 s, followed by primer annealing at 55°C for 30 s and primer extension at 72°C for 60 s. After 24 cycles of reaction, an extension step was followed at 72°C for 2 min. The PCR products were detected with 2% agarose gel electrophoresis. The bright and single PCR products were sent to Genesky Biotechnologies Inc. (Shanghai) for sequencing by using the Illumina NovaSeq second-generation high-throughput sequencing platform with a 2 × 250 bp paired-end sequencing strategy.

Firstly, the quality of the soil samples’ genomic DNA was detected, and the detection area of qualified sample was amplified by high-fidelity PCR. Primers with Index sequences were used to introduce specific tag sequences to the end of each sample library by high-fidelity PCR. The Qubit was used to accurately quantify the library and the samples were mixed in corresponding proportions. The size of the inserted fragment of the sequencing library was detected by Agilent 2100 Bioanalyzer to confirm non-specific amplification between 120 and 200 bp, and the concentration of the sequencing library was accurately quantified. The library was sequenced by 2 × 250 bp paired-end sequencing strategy, and then bioinformatics analysis was performed.

### 2.5 Myxobacteria assemblages

Raw 16S rRNA sequence data were removed of chimeras using software TrimGalore and FLASH2 (Magoc and Salzberg, 2011), then clustered into operational taxonomic units (OTUs) at the 97% sequence similarity. The most abundant sequence from each OTU was selected as the representative sequence for that OTU. Mothur was used to calculate the values for different random samples and the R language tool was used to create the sample diluency curves (Amato et al., 2013). The OTU diversity was analyzed using QIIME v1.9.02 (Caporaso et al., 2010) following the standard workflow, and the α diversity index were calculated using the mothur program v.1.30.1 (Schloss et al., 2009). Taxonomy was assigned to the OTU representative sequences (phylotypes) using BLAST (version 2.2.30+, Altschul et al., 1997), RDP (Cole et al., 2009), and SILVA (Quast et al., 2013) against a custom reference collection of 16S rRNA gene sequences, which was compiled from cultured or otherwise well-characterized species in GenBank. Significant positive and negative correlations between OTUs were determined individually by support of Pearson’s correlation measures.

### 2.6 Statistical analyses

All statistical analyses were done with the software IBM SPSS Amos 19.0. The significant difference was calculated using Tukey’s test under *P* < 0.05.

## 3 Results

### 3.1 The physiochemical properties of soil samples

In this study, pH value, the contents of water, organic matter, available phosphorus, hydrolyzed nitrogen, and available potassium of 133 soil samples collected in the Inner Mongolia Autonomous Region were determined and the results were shown in Supplementary Table 2.

Compared with the grading standards of soil nutrient indicators in the second national soil survey in China (Supplementary Table 3), the results were shown in Table 1. Among the 133 soil samples, 46.27% were neutral soils, 35.07% were slightly alkaline to strong alkaline soils, and 17.17% were slightly acidic to strong acidic soils. A total of 89.54% of the soil samples were below 16% in a state of light drought to heavy drought, water content of 7.19% soil samples was in a wet state, and 3.74% of the soil samples were at an appropriate level of water content. For the content of TOM, 67.17% of soil samples were at a low to very low level, 20.89% were at high to extremely high level, and 11.94% were at medium level. The content of available phosphorus was distributed evenly, with a certain proportion in each grade. Of which, 55.97% of soil samples were at low to extremely low level, 25.37% were at high to very high level, and 18.66% were at medium level. As for the content of available potassium, 76.12% of soil samples were at low to extremely low level, 12.69% at medium level and 11.19% at high to very high level. However, the content of hydrolyzed nitrogen was generally in a state of deficiency, with 80.59% of soil samples at a low to extremely low level. These results indicated that soil fertility was generally poor in Inner Mongolia.

**TABLE 1:** pH and nutrient grade distribution of soil samples.

Level	Percentage of each level (%)					
	**Water content**	**pH**	**Organic matter**	**Available phosphorus**	**Hydrolyzed nitrogen**	**Available potassium**
I		1.49	11.19	16.42	10.45	5.22
II	7.19	33.58	9.70	8.95	2.99	5.97
III	3.74	46.27	11.94	18.66	5.97	12.69
IV	17.16	8.96	43.28	22.39	21.64	22.39
V	31.34	8.21	20.90	14.18	29.85	11.19
VI	41.04	1.49	2.99	19.40	29.10	42.54

### 3.2 The relative abundance of myxobacteria

The relative abundance of myxobacteria in soil samples from different groups was determined by qPCR. In order to avoid the influence of primer bias on the experimental results, genomic DNA of *Myxococcus xanthus* BM30, *Sorangium cellulosum* S-C12, and *Nannocystis exedens* DSM14640 were used as templates in this study. The universal primers 1369F/1541R for bacteria and W5/802R for myxobacteria were used for amplification and standard curves were drawn (Supplementary Figure 1). The results were shown in Table 2.

**TABLE 2:** The relative abundance of myxobacteria in soil samples from different groups.

The relative abundance of myxobacteria calculated by drawing standard curves using the DNA sequences of different suborders as amplification templates (%)		I	II	III	IV	V
Genomic DNA of *Myxococcus xanthus* BM30 as template	Range	0.25–3.19	1.57–4.45	1.17–4.47	0.01–3.92	0.91–6.40
	Means ± SE	2.03 ± 0.72	3.04 ± 0.77	2.47 ± 0.74	2.57 ± 0.73	2.35 ± 1.06
Genomic DNA of *Sorangium cellulosum* S-C12 as template	Range	0.02–3.04	0.81–6.08	0.35–8.78	0.00–10.28	0.15–7.02
	Means ± SE	1.08 ± 0.77	3.81 ± 1.67	2.17 ± 2.22	2.37 ± 1.90	1.53 ± 1.44
Genomic DNA of *Nannocystis exedens* DSM14640 as template	Range	0.06–4.93	1.51–8.67	0.87–8.69	0.00–8.31	0.52–14.27
	Means ± SE	2.27 ± 1.16	4.93 ± 1.90	3.32 ± 1.92	3.56 ± 1.43	3.03 ± 2.51

*Myxococcus xanthus* BM30 means the relative abundance of myxobacteria was calculated according to the standard curve amplified by different primers using *M. xanthus* BM30 as the template. *S. cellulosum* S-C12 means the relative abundance of myxobacteria was calculated according to the standard curve amplified by different primers using *S. cellulosum* S-C12 as the template. *N. exedens* DSM14640 means the relative abundance of myxobacteria was calculated according to the standard curve amplified by different primers using *N. exedens* DSM14640 as the template.

Based on the standard curves drawn with the genomic DNA of *M. xanthus* BM30 as the amplification template, the relative abundance of myxobacteria was calculated to range from 0.01% to 6.40% and the average relative abundance was 2.45%. The average relative abundance of myxobacteria in soil samples from group II was the highest (3.04%), followed by group IV. The average relative abundance of myxobacteria in soil samples from group III and group V was relatively close (2.47% and 2.35%, respectively), while the average relative abundance of myxobacteria in soil samples from group I was the lowest (only 2.03%).

The relative abundance of myxobacteria was calculated from the standard curves drawn using the genomic DNA of *S. cellulosum* S-C12 as the amplification template, ranging from 0.00% to 10.28% and the average relative abundance was 2.06%. The average relative abundance of myxobacteria in soil samples from group II was the highest (3.81%). The average relative abundance of myxobacteria in soil samples from group IV and group III was relatively close, 2.37% and 2.17%, respectively. The average relative abundance of myxobacteria in soil samples from group I was the lowest (1.08%), while that in group V was slightly higher than that in group I (1.53%).

Based on the standard curves drawn with the genomic DNA of *N. exedens* DSM14640 as the amplification template, the relative abundance of myxobacteria was calculated to range from 0.00% to 14.27% and the average relative abundance was 3.30%. The average relative abundance of myxobacteria in soil samples from group II was the highest (4.93%). The average relative abundance of myxobacteria in soil samples from group IV, group III, and group V was close and more than 3%, which were 3.56%, 3.32%, and 3.03%, respectively. The average relative abundance of myxobacteria in soil samples from group I was the lowest, only 2.27%.

It can be seen that no matter which genus of myxobacteria was used as the amplification template for the standard curves, the average relative abundance of myxobacteria in soil samples from group II was the highest, followed by group IV. The average relative abundance of myxobacteria in soil samples from group III was the middle, group I was the lowest, and group V was slightly higher than group I. The results showed that the average relative abundance of myxobacteria in the soil samples from the central part of Inner Mongolia was higher than that in the eastern and western parts.

### 3.3 16S rRNA gene sequencing of myxobacteria

High-throughput sequencing of 16S rRNA gene was used to reveal the community structure of myxobacteria. A total of 20,265,692 quality-filtered and chimera-checked 16S rRNA gene sequences were obtained with an average length of 231 bp across all samples. The 16S rRNA gene sequences were submitted to the GenBank database, and the accession number was PRJNA1112089. The sequences with a similarity greater than 97% were clustered into the same OTU, and a total of 5,595 OTUs of myxobacteria were detected. A part of sequence was randomly selected from each sample, and then the number of OTUs corresponding to this part of the reads in the sample was counted. Through the results of multiple random sampling, the rarefaction curves were constructed based on the number of sequences selected and the number of OTUs they could represent. As shown in Supplementary Figure 2, the number of OTU in each sample increased with the increase of sequencing depth and then leveled off, indicating that the amount of sequencing data was reasonable and could better reflect the community structure and diversity of myxobacteria in soil samples.

### 3.4 Genetic diversity of myxobacteria community

**FIGURE 1:**
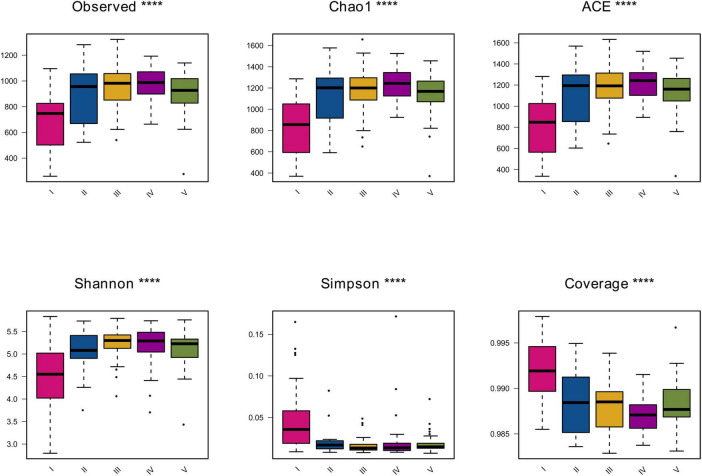
The α diversity index box diagram. The figure shows the six diversity indices of each group. The abscissa represents different groups, and the ordinate represents the community diversity index value of the group. Different groups are distinguished by different colors. *⁣*⁣**P-value < 0.05 as the screening threshold for difference significance between groups, the greater the difference between groups, the greater the number of *.

The α diversity indices can be used to measure the species richness and diversity of each sample in the community ecology. The indices for evaluating the richness and diversity of myxobacteria in each sample were calculated and the box diagram of α diversity indices among different groups was drawn. The results were shown in Figure 1.

The indices for calculating the bacterial community richness include Observed, Chao1 and ACE. As shown in Figure 1, it was found through calculation of three different indices that the richness indices of soil samples in the fourth group were slightly higher than those of soil samples in the second, third and fifth groups. And, the richness indices of soil samples in the second, third and fifth groups were similar to each other in terms of myxobacteria diversity level, while the Observed, Ace, and Chao1 values of soil samples in the first group were always lowest than that of other groups. It can be seen that the community richness of myxobacteria in the first group of samples is significantly lower than that of the other four groups.

The indices for calculating the diversity of the bacterial community include Shannon, Simpson, and Coverage. As shown in Figure 1, by comparing the overall Shannon and Simpson indices among the groups, it was found that they showed similar results in reflecting the diversity of the sample groups. The diversity of the samples in the first group was the lowest, and the overall diversity of the other four groups was basically similar, all of which were higher than that of the first group. All coverage values were over 98%, suggesting the sequencing depth was sufficiently high to represent the real diversity and composition of myxobacteria communities. Among them, the coverage of samples in the first group was the highest, and the coverage of the other four groups was also high but lower than that of group I. In conclusion, the samples in groups II, III, IV, and V had high richness and diversity, but their coverage was lower than that of group I.

### 3.5 Composition of myxobacteria community

**FIGURE 2:**
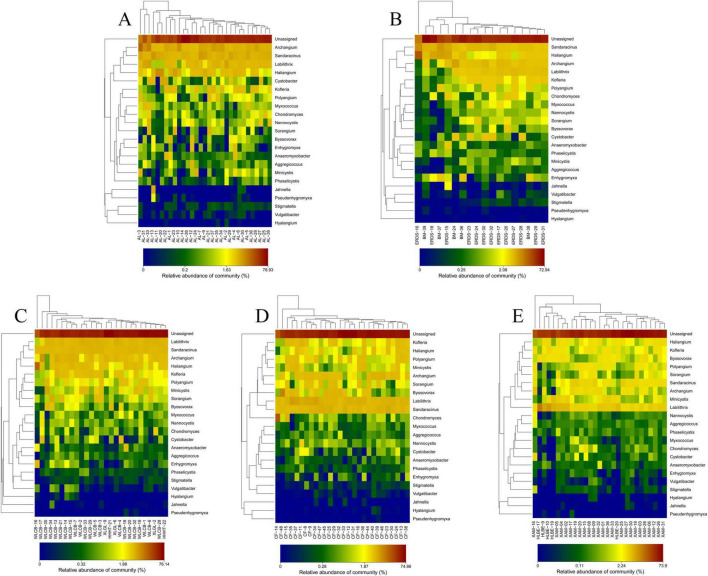
Heatmap of communities of myxobacteria at the genus level. The abscissa is the sample, and the ordinate is the 100 species with the highest abundance at the genus level. The colors in the figure represent the relative abundance of species, and the gradual change of color from blue to red indicates the change of relative abundance from small to large. **(A)** Group I, **(B)** group II, **(C)** group III, **(D)** group IV, **(E)** group V.

In this analysis, the top 100 myxobacteria species with the highest relative abundance were selected by default to draw the heatmap, and the results were shown in Figure 2. The composition of myxobacteria communities in the five groups of soil samples distributed in different regions was similar to each other, including 3 suborder, 10 families, and 22 genera. At the family level, the five groups all contained Polyangiaceae, Cystobacteraceae, Labilitrichaceae, Sandaracinaceae, Haliangiaceae, Nannocystaceae, Kofleriaceae, Myxococcaceae, Phaselicystidaceae, and Vulgatibacteraceae. At the genus level, the five groups all contained *Labilitrix*, *Sandaracinus*, *Archangium*, *Haliangium*, *Kofleria*, *Minicystis*, *Polyangium*, *Byssovorax*, *Sorangium*, *Chondromyces*, *Cystobacter*, *Nannocystis*, *Myxococcus*, *Enhygromyxa*, *Aggregicoccus*, *Anaeromyxobacter*, *Phaselicystis*, *Jahnella*, *Stigmatella*, *Vulgatibacter*, *Pseudenhygromyxa*, and *Hyalangium*.

In addition, the species with high, medium and low abundance of myxobacteria in the five groups were different. The relative abundances of *Labilitrix*, *Sandaracinus*, *Archangium*, and *Haliangium* in the groups I–III were high and more than 4% (Supplementary Table 4). In the fourth and fifth groups, the species with relative abundance more than 4% were only *Sandaracinus* and *Labilitrix*. The relative abundances of *Jahnella*, *Stigmatella*, *Vulgatibacter*, *Pseudenhygromyxa*, and *Hyalangium* in the five groups were all low and less than 0.2%. The rest of myxobacteria were medium abundance species. Among them, the dominant myxobacteria in the first group was *Archangium*, the dominant myxobacteria in the second group was *Sandaracinus*, and the dominant myxobacteria in the third, fourth and fifth groups were all *Labilitrix*. It can be seen that *Labilitrix*, *Sandaracinus*, *Archangium*, and *Haliangium* were the dominant species of myxobacteria in Inner Mongolia. In addition, the fraction of unknown taxon unassigned in the five groups were dominant taxa, and their relative abundances were more than 59%. This suggests that many unknown myxobacteria exist in soils. Thus, the detailed information on those rare myxobacteria and unknown myxobacteria requires deeper sequencing and mining in the future.

### 3.6 Analysis of species differences of myxobacteria among different groups

**FIGURE 3:**
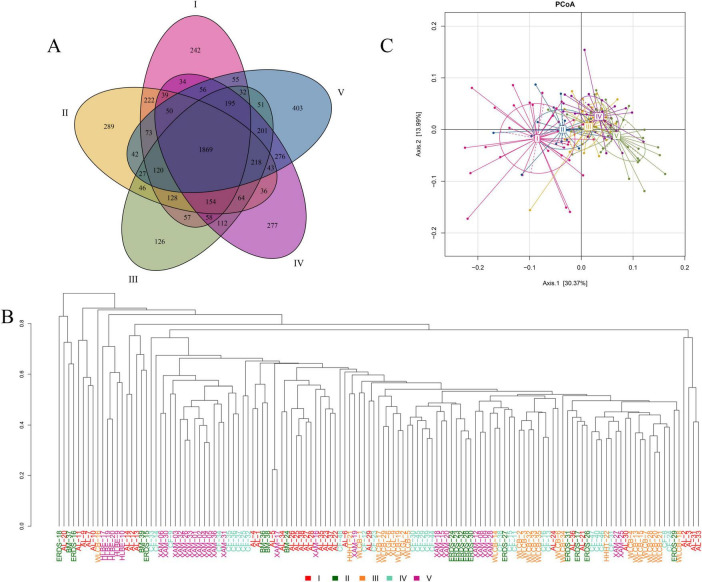
Analysis of species differences of myxobacteria among different groups. **(A)** Venn diagram of myxobacteria species (OTUs) composition. **(B)** Hierarchical clustering tree based on the beta diversity of distance. Samples in different groups were distinguished by different colors. The length of the branches represented the distance among samples. The closer the branches were, the more similar the species composition of the samples was. **(C)** PCoA analysis of myxobacteria species (OTUs) at the genus level. The abscissa (Axis.1) and the ordinate (Axis.2) were the two main components with the largest interpretation of the difference among samples. The scale was the relative distance and had no practical meaning. Each point in the figure represented a sample and the different colors of the points indicated the groups the samples belonged to. The closer the points were, the more similar the samples were.

Samples in different groups have certain characteristics and commonalities. Based on the OTU abundance table, a Venn diagram was drawn to screen the unique OTUs in each group of samples and the common OTUs among groups. The results were shown in Figure 3A. The OTUs of myxobacteria species in the samples of groups I to V were 3,384, 3,420, 3,458, 3,682, and 3,711, respectively. The total number of OTUs of myxobacteria species was 5,595, of which 1,869 OTUs were shared by the five groups, accounting for 33.40%. The unique OTUs of groups I to V were 242, 289, 126, 277, and 403, accounting for 4.33%, 5.17%, 2.25%, 4.95%, and 7.20% of the total number of OTUs, respectively. It can be seen that the differences in the composition of myxobacteria among the five groups of samples are small.

In order to describe the similarity and difference of species as a whole and compare the similarity and difference of the sample species among groups, this study calculated the Bray–Cruits dissimilarity matrix among samples based on the OTU abundance, and then carried out the average linkage hierarchical clustering on the samples and drew the samples cluster tree diagram (Jiang et al., 2013). The closer the branches are, the more similar the species composition of the samples is. As shown in Figure 3B, the species composition of some samples in the same group was basically similar, but the species composition of some samples was quite different, and the species composition of samples in different groups may also be similar situation. For example, XAM-1, XAM-2, XAM-3, XAM-4, XAM-5, XAM-6, XAM-26, XAM-30, XAM-31, XAM-32, XAM-33, and XAM-36 in the group V and CF-9, CF-34, CF-35, CF-36, and CF-37 in the group IV were all on the same branch, but they are in the same group as XAM-8, XAM-9, XAM-12, XAM-15, XAM-18, XAM-20, and XAM-28 were on different branches and were far apart. Most samples were clustered according to the same or similar sampling locations. There were some exceptions, which may be related to specific environmental parameters. It can be preliminarily inferred that the distribution of soil has an impact on the species composition of the samples, but it was not the only factor affecting the species composition. In addition, soil type also had an effect on the species composition of the samples. For example, soil samples CF-29, CF-30, CF-39, CF-44, and CF-45 were Cinnamon soils and on the same branch. Soil samples AL-25, AL-26, AL-35, AL-37, and AL-38 were gray desert soils and on the same branch.

By default, principal coordinate analysis (PCoA) was performed with weighted unifrac distance that comprehensively considered the species and their abundance differences in the samples, and the results were shown in Figure 3C. The distance between the sample points in different groups was separated but not obvious, and the distance increased with the distribution of samples from west to east. The distance between group I and group V was relatively long, indicating a low similarity, while the distance between group III and group IV was close, indicating a high similarity of myxobacteria community structure. The above analysis showed that there were differences in myxobacteria community structure in the samples among different groups, and the degree of difference increased with the increase of longitude difference.

**FIGURE 4:**
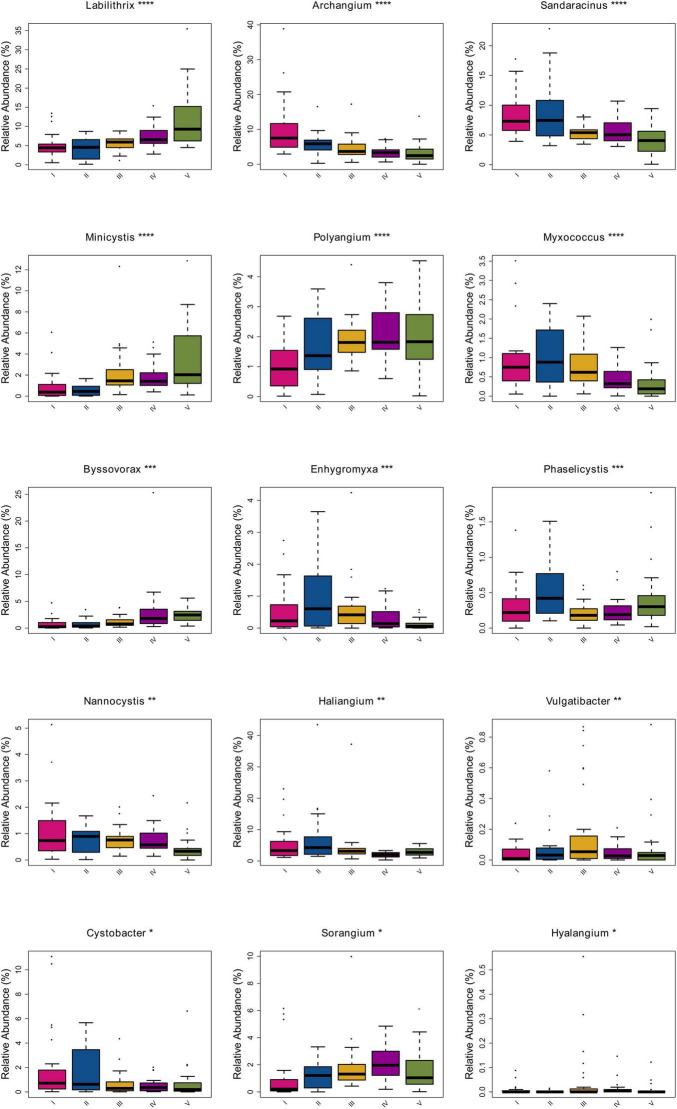
A comparison box diagram of difference species in ANOVA. The box diagram mainly was a method to describe the data by using five statistics in the data: the minimum value, the first quartile (25%), the median (50%), the third quartile (75%), and the maximum value. It can also roughly see the degree of sample dispersion within a group and the difference in species abundance among different groups. Different colors represented different groups of samples, the abscissa represented the different species at the classification level, and the ordinate represented the relative abundance value of the species. *, **, ***, *⁣*⁣**P-value < 0.05 as the screening threshold for difference significance between groups, the greater the difference between groups, the greater the number of *.

With *P*-value < 0.05 as the screening threshold for difference significance, ANOVA analysis of variance was used to find out the species with significant differences in the five groups and draw a box diagram. The results were shown in Figure 4. The species with significant differences in the relative abundance of the genus level among sample groups were *Labilitrix*, *Archangium*, *Sandaracinus*, *Minicystis*, *Polyangium*, and *Myxococcu*s, followed by *Byssovorax*, *Enhygromyxa*, and *Phaselicystis*, then followed by *Nannocystis*, *Halangium*, and *Vulgatibacter*, and finally followed by *Cystobacter*, *Sorangium*, and *Hyalangium*. Among them, the relative abundances of *Labilitrix*, *Minicystis*, *Polyangium*, and *Byssovorax* gradually increase from west to east, while the relative abundances of *Archangium*, *Sandaracinus*, and *Cystobacter* gradually decrease from west to east. The relative abundances of *Myxococcu*s, *Enhygromyxa*, *Phaselicystis*, *Nannocystis*, and *Halangium* in the second group of samples were higher than those in the other groups. The relative abundance of *Vulgatibacter* in the third group of samples was higher than it in the other groups. The relative abundance of *Sorangium* in the third group of samples was higher than it in the other groups.

### 3.7 Correlation analysis between myxobacteria community structure and environmental factors

**FIGURE 5:**
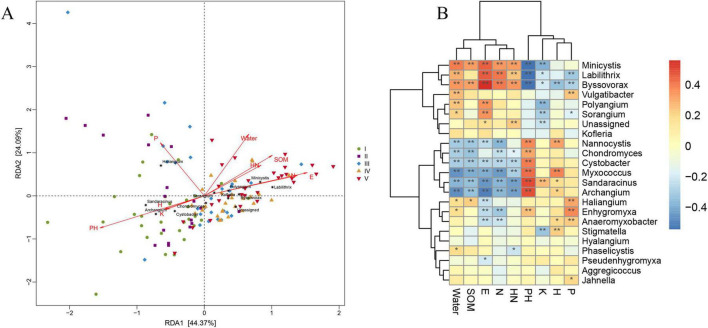
Correlation analysis between myxobacteria community structure and environmental factors. **(A)** Redundancy analysis of the relatedness between community structure of myxobacteria and soil physic-chemical properties. The spatial distance of the sample points represented the distance between samples, and the projected distance of vectors from the sample points to the environmental factors represented the degree to which the samples were affected by environmental factors. The sample points were perpendicular to the environmental factors, and the projection points were in the direction of the arrow, indicating that there was a positive correlation between the environmental factor and the sample, and vice versa. The angle between the species and the environmental factors represented the positive and negative correlation between the species and the environmental factors. The acute angle represented a positive correlation, the obtuse angle represented a negative correlation, and the right angle represented that there was no correlation between the two. **(B)** Heatmap of the correlation between myxobacteria species and environmental factors. The abscissa was different environmental factors, and the ordinate was the species with the highest abundance at the genus level. Different colors indicated the size of the correlation coefficient between pairs. The darker the blue was, the stronger the negative correlation, and the darker the orange was, the stronger the positive correlation. The “*” on the square indicated the significance *P* < 0.05, and the “**” indicated the significance *P* < 0.01.

The relationship between the myxobacteria community structure at the genus level and physical and chemical properties of soil samples in Inner Mongolia was analyzed by RDA (Sheik et al., 2012), and the results were shown in Figure 5A. The latitude and longitude of the sampling point and the soil pH, contents of water, available phosphorus and organic matter had a greater impact on the myxobacteria community structure of samples, while the altitude of the soil sample and the contents of available potassium and alkaline nitrogen in the sample had a relatively small impact. Among them, the effect of pH on the myxobacteria community structure of samples was the largest, and the effect of available potassium on the samples was the smallest. The myxobacteria community structure of most samples in the first and second groups were positively correlated with the soil pH, altitude of the sampling points, contents of available potassium and available phosphorus, and negatively correlated with the latitudes and longitudes of the sampling points, contents of water, organic matter, and hydrolyzed nitrogen of the soil. The myxobacteria community structure of most samples in the third, fourth and fifth groups were positively correlated with the latitudes and longitudes of the sampling points, contents of water, organic matter, and hydrolyzed nitrogen of the soil, and negatively correlated with the soil pH, altitude of the sampling points, contents of available potassium, and available phosphorus.

Based on Pearson correlation analysis of the correlation between each species and environmental factors, it is possible to evaluate which species and the direction of impact the environmental factors specifically affect. With the correlation coefficient of —cor— > 0.3 and *P* < 0.05 as the significance screening threshold, we can screen the species that are significantly correlated with environmental factors. In order to visually show the relationship between species and environmental factors, a correlation heatmap was drawn (Figure 5B). *Minicystis*, *Labilithrix*, and *Byssovorax* were positively correlated with the latitude and longitude of the sampling point, contents of water and hydrolyzed nitrogen, and negatively correlated with soil pH and content of available potassium. *Minicystis* and *Byssovorax* were also positively correlated with content of organic matter, and *Labilithri*x and *Byssovorax* were also negatively correlated with content of available phosphorus. *Cystobacter*, *Myxococcus*, *Sandaracinus*, and *Archangium* were all negatively correlated with the latitudes and longitudes of the sampling points, contents of water, organic matter, and hydrolyzed nitrogen of the soil, and positively correlated with the altitude of the sampling points and the pH value of the soil. *Chondromyces* was negatively correlated with the latitude of the sampling point, and the contents of water, organic matter, and hydrolyzed nitrogen, while positively correlated with the pH value of the soil. *Nannocystis* was negatively correlated with the latitude and longitude of the sampling point as well as contents of water and organic matter content, and positively correlated with the altitude of the sampling point and the pH value of the soil. The results indicated that the distribution and nutrient content of the soil samples determine the relative abundance of the myxobacteria community. And these strong correlations suggest that myxobacteria were actively involved in the nutrient cycle within soils.

### 3.8 Predictive analysis of myxobacteria community function

**FIGURE 6:**
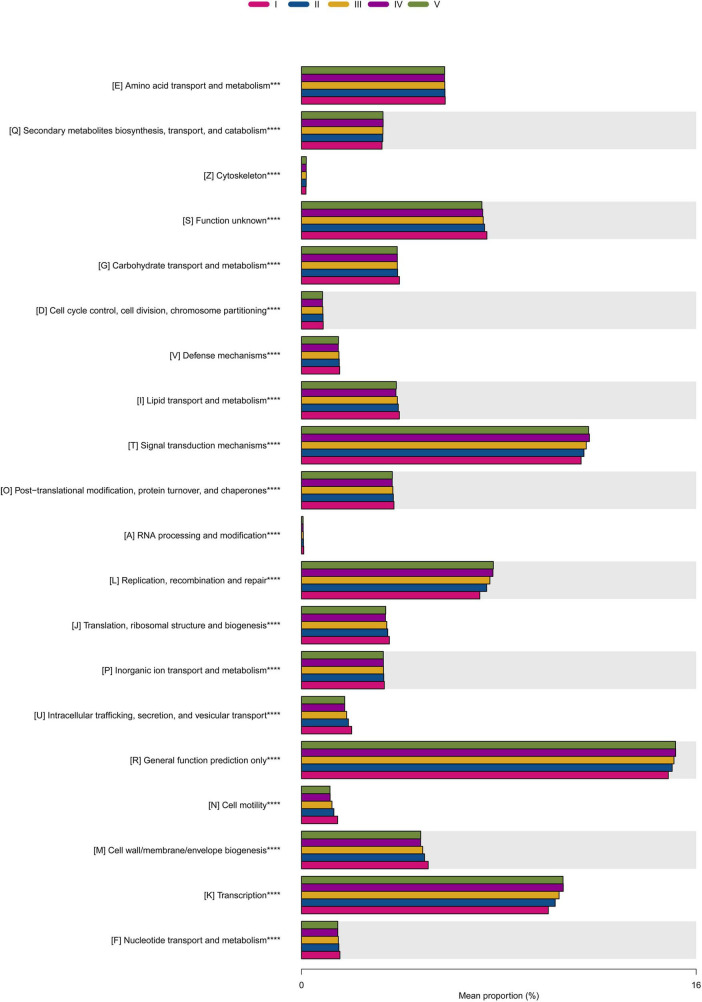
PICRUSt functional gene prediction of myxobacteria community. Each color represented a set of samples. The bar graph represented the pathways with significant differences in abundance in the five groups of samples, and their average relative abundance. ***, *⁣*⁣**P-value < 0.05 as the screening threshold for difference significance between groups, the greater the difference between groups, the greater the number of *.

In order to analyze the changes in the functional composition of myxobacteria among the five groups, the PICRUSt software was used to predict and analyze myxobacterial functions. The results were shown in Figure 6. A total of 20 COG functional groups were obtained in this study, and their relative abundance varied with different samples. They were mainly involved in the amino acid transport and metabolism, secondary metabolites biosynthesis, transport, and catabolism, carbohydrate transport and metabolism, lipid transport and metabolism, signal transduction mechanisms, post-translational modification, protein turnover, and chaperones, replication, recombination and repair, translation, ribosomal structure and biogenesis, inorganic ion transport and metabolism, cell wall/membrane/envelope biogenesis, and transcription. Some studies have shown that myxobacteria have inhibitory activity against a variety of plant pathogenic microorganisms (Wu et al., 2021). It was also found in this article that some myxobacteria participated in the biosynthesis of antibiotics, indicating that they may be involved in the prevention and control of plant diseases.

## 4 Discussion

As a phylogenetically coherent and distinctive group, members of the order Myxococcales have been divided into three suborders, 11 families, 30 genera, and 66 species, mainly based on morphological properties of cells and sequences analysis of the 16S rRNA gene. Myxobacteria are typically soil-living, broad-spectrum-predatory, Gram-negative δ-proteobacteria that are widely distributed in most climate zones worldwide (Xu et al., 2021; Hoffmann et al., 2018). Myxobacteria are essential soil bacteria. They live in a wide range of environments, such as temperate zones, activated sludge (Karst et al., 2018), rain forests, arctic tundra, deserts, acidic soils, and marine and other saline environments (Mohr, 2018), and are distributed throughout the world but are predominantly found in soils (Muñoz-Dorado et al., 2016). In addition to a minority of type strains, the majority of non-type strains have been isolated from diverse ordinary and extreme habitats, and preserved in multiple culture collections or research teams, for example, more than 9,000 myxobacteria were collected by Reichenbach and Höfle (Hoffmann et al., 2018). Compared to many in-depth studies on the physiological basis of social behavior and on action mechanisms of secondary metabolites of myxobacteria over the last several decades, much less attention has been paid to their taxonomic diversity and biogeographic distribution across heterogeneous environments. The detailed relationships between taxa and different environments and the distribution patterns of myxobacteria have not yet been thoroughly investigated.

### 4.1 The physiochemical properties of soil samples

In this study, a total of 133 soil samples were collected from west to east in the Inner Mongolia Autonomous Region, China. According to the analysis and statistics of the soil parameters, it can be seen that the soil samples in Inner Mongolia were basically neutral and alkaline, and the water content of the soil was very low, which was generally in a dry state. The content of hydrolyzed nitrogen was in the state of lack as a whole. The contents of organic matter and available potassium in more than half of the soil samples (67.17% and 76.12%, respectively) were in a state of lack, while the content of available phosphorus was relatively high, but 55.97% of the soil samples were below the medium level. The suitability of single element could not improve the overall fertility of nutrients. According to the analysis of soil nutrients in Guanzhong area of Shanxi Province (Zhao, 2015), it can be seen that the contents of organic matter, hydrolyzed nitrogen, available phosphorus, and available potassium in this area were respectively in the fifth, fourth, third, and second level of the soil nutrient classification standard, that is, except for organic matter in a state of deficiency, other contents were at the suitable level or above suitable level. Fu (2005) analyzed the soil resource of the vegetable base in Zhengzhou, Henan Province and found that the soil fertility in this suburb was medium, and the contents of other nutrients were at a medium level except for the low content of available phosphorus. In comparison, it can be seen that the soil in Inner Mongolia was severely dry and the soil fertility level was low, which can also explain a series of ecological environmental degradation problems in this region, such as the severe sandstorm, desertification, and vegetation degradation.

### 4.2 Diversity and community structure of myxobacteria

Some scientists have found myxobacteria in soil samples collected in the Antarctic, which do not survive in laboratory conditions. *Myxococcus* and *Nannocystis* were first discovered in the environment of 6–8°C in the Alps, and some studies have also found traces of *Myxococcus* in the high-temperature desert soil of Arizona, USA. Due to the slow growth of myxobacteria, the difficulty of purification and the special method of separation and purification (Mohr et al., 2018b; Livingstone et al., 2020), it cannot be achieved through one or two operations. In addition, with regard to the identification and classification of myxobacteria, common isolation and purification methods may not be accurate enough and/or can only analyze a limited number of species in samples from complex environments, so the impact of soil types on myxobacteria communities cannot be fully understood (Huhe et al., 2017). Therefore, other better methods are needed to study and analyze myxobacteria. In this study, the quantitative assessment of the abundance of myxobacteria in the soil sample were performed by qPCR. We used *M. xanthus* BM30, *S. cellulosum* S-C12, and *N. exedens* DSM14640 as amplification templates to draw standard curves, and the average relative abundances of myxobacteria in soil samples calculated by them were 2.45%, 2.06%, and 3.30% respectively, which was lower than the relative abundance of myxobacteria surveyed by Zhou et al. (2014) (4.10%). Through a comparative analysis of the EMP data and public information, Wang et al. (2021) determined that myxobacteria account for 2.34% of the total bacterial OTUs, and are one of the most diverse bacterial groups on Earth. The diversity of myxobacteria resources in Inner Mongolia was analyzed by NovaSeq high-throughput sequencing technology, and it was found that the myxobacteria community of soil in this area included 10 families and 22 genera, containing almost all of the cultivated myxobacterial families or genera, among which the dominant genera were *Labilitrix*, *Sandaracinus*, *Archangium*, and *Haliangium*. These results suggested that myxobacteria was a dominant group in the bacterial community in the soil sample in terms of both population and species numbers.

The species composition of myxobacteria among different groups was analyzed, and it was found that the distribution of soil had an impact on the species composition of the samples, but it was not the only factor that affected the species composition. The species with significant differences in relative abundance among the five groups of samples were *Labilitrix*, *Archangium*, *Sandaracinus*, *Minicystis*, *Polyangium*, and *Myxococcus*. Among them, the relative abundances of *Labilitrix*, *Minicystis*, and *Polyangium* gradually increased from west to east, while the relative abundances of *Archangium* and *Sandaracinus* gradually decreased from west to east. Gaspari et al. (2005) isolated 190 myxobacteria strains from Israel, which belonged to the 12 described genera of myxobacteria, including *Myxococcus*, *Corallococcus*, *Angiococcus*, *Archangium*, *Cystobacter*, *Melittangium*, *Stigmatella*, *Sorangium*, *Polyangium*, *Chondromyces*, *Haploangium*, and *Nannocystis*. The majority of their isolated strains belonged to the genus *Myxococcus* and *Corallococcus* (Gaspari et al., 2005). Compared with the genera found in this study, 16S rRNA gene sequences of *Labilitrix*, *Sandaracinus*, *Haliangium*, *Kofleria*, *Minicystis*, *Byssovorax*, *Enhygromyxa*, *Aggregicoccus*, *Anaeromyxobacter*, *Phaselicystis*, *Jahnella*, *Vulgatibacter*, *Pseudenhygromyxa*, and *Hyalangium* were only detected in the soil samples of Inner Mongolia. The number of myxobacteria genera isolated by them were far less than the results in this study, and the dominant myxobacteria were also different from the dominant genera in Inner Mongolia. This may be due to the community structure of myxobacteria in the two regions was different. It was also possible that they used traditional methods to isolate myxobacteria of Israel, but this study used high-throughput sequencing technology to analyze diversity of myxobacteria resources in Inner Mongolia, so more species of myxobacteria can be found. Wang et al. (2021) employed a 10,000-sample subset (5,000 sequences were randomly selected from each sample) to evaluate the distribution and diversity of the myxobacterial community on Earth and determined 21 myxobacterial genera, among which all the other genera of myxobacteria were detected in Inner Mongolia except *Pyxidicoccus*. *Pseudenhygromyxa* and *Hyalangium* were only detected in Inner Mongolia, and their gene sequences were not found in Wang et al.’s (2021) research. They found that at the genus level, *Haliangium* of Haliangiaceae, *Cystobacter* of Cystobacteraceae, and *Chondromyces* of Polyangiaceae were the top 3 among the 21 myxobacterial genera in the environment, which was different from the dominant genera in Inner Mongolia. This may be because the research scope of Wang et al. (2021) was different from that of this study, and their research scope was broader. However, this study only focused on the excavation of myxobacteria resources in Inner Mongolia. These results also showed that the soil microhabitats in different areas were different, which determined the differences of myxobacteria community structure. In China, Li et al. (2000) isolated hundreds of myxobacteria from samples collected in more than 20 provinces and cities in China around 2000, belonging to 10 different genera. Zhang et al. (2012) collected 25 samples from saline-alkaline soils of Akesu in Xinjiang. In total 58 strains were isolated, and identified as belonging to 6 different genera, i.e., *Myxococcus*, *Cystobacter*, *Corallococcus*, *Sorangium*, *Nannocystis*, and *Polyangium* of Myxococcales. Zhang et al. (2018) isolated 64 strains from 84 samples in Guangdong, The pure cultures could be classified into 7 genera *Myxococcus* (32 strains), *Corallococcus* (15 strains), *Cystobacter* (2 strains), *Polyangium* (5 strains), *Archangium* (7 strains), *Pyxidicoccus* (5 strains), and *Hyalangium* (1 strain). The number of myxobacteria genera isolated by them were far less than the results in this study. The 16S rRNA gene sequences of *Labilitrix*, *Sandaracinus*, *Haliangium*, *Kofleria*, *Minicystis*, *Byssovorax*, *Chondromyces*, *Enhygromyxa*, *Aggregicoccus*, *Anaeromyxobacter*, *Phaselicystis*, *Jahnella*, *Stigmatella*, *Vulgatibacter*, and *Pseudenhygromyxa* were only detected in soil samples from Inner Mongolia by high-throughput sequencing technology, but *Corallococcus* and *Pyxidicoccus* were not detected in soil samples from Inner Mongolia. For the current research on the diversity of myxobacteria, in addition to different research methods, the samples collected in most of the studies were also less. We collected 133 soil samples from the whole region of Inner Mongolia. The research on such a large range of samples was more representative.

### 4.3 Ecological function of myxobacteria

The Gram-negative gliding myxobacteria are characterized by their sophisticated multicellular lifestyle (Wang C. L. et al., 2020) and can produce abundant secondary metabolites with complex structure and biological activity. It is a kind of microbial community that needs to be developed urgently and has wide application potential in agriculture, biomedicine, and environmental protection. For example, myxobacteria are able to produce a variety of bioactive secondary metabolites and are one of the most important bacterial resources for the discovery of novel antibiotics; more than 100 new carbon skeleton metabolites and over 600 derivatives have been identified from myxobacterial strains (Hoffmann et al., 2018; Landwehr et al., 2016). Some myxobacteria produce diverse carotenoids (Moraleda-Muñoz et al., 2005), degrade 2-chlorophenol (Sanford et al., 2002), and reduce uranium (VI) (Wu et al., 2006). In terms of medicine, Guo (2007) found that *Stigmatella* sp. has strong inhibitory effect on tumor cell lines B16 and SGC7901. In terms of ecological environment, *Polyangium parasitium* can parasitize on Cladophora and cause the algae to die (Zhao and Liu, 1996). In agriculture, Wu (2018) found that *M. xanthus* and *Myxococcus fulvus* have antagonistic effects on *Phytophthora infestans* and other plant pathogens. In addition, myxobacteria are able to prey on many other bacteria and fungi, and are micropredators that regulate bacterial communities in agricultural land (Wang W. H. et al., 2020). Some myxobacteria can prevent and control cucumber Fusarium wilt by regulating the soil microbial community (Ye et al., 2020). In industrial production, *S. cellulosum* can convert cellulose into biofuel, providing new energy options (Yuan et al., 2015). Therefore, it is of great significance to study the diversity and distribution of myxobacteria resources for the development and utilization of myxobacteria in the next step.

### 4.4 Response mechanism of myxobacterial community structure and diversity to soil environment

The 133 soil samples were divided into five groups from west to east according to the different geographical locations of the soil. The richness and diversity index of the myxobacteria community among different groups were compared. It was found that the richness and diversity of the samples in groups II, III, IV, and V were higher than those in group I, but the coverage was lower than that of the first group of samples. The results of redundant analysis showed that the latitude and longitude of the sampling site, as well as the pH value, contents of water, available phosphorus, and organic matter have a greater impact on the samples, while the altitude of the soil samples and the contents of available potassium and alkaline nitrogen had a relatively small impact. Wang C. L. et al. (2020) found that soil pH and bacterial diversity have significantly relationship with myxobacterial community. Wang et al. (2019) believed that soil pH was the most important property that derived myxobacterial abundance and cell density in red soil. Soil pH as a driver of the soil bacterial and fungal community has also been reported in other studies (Bahram et al., 2018; Sun et al., 2015). These findings were consistent with the results of this study. Zhou et al. (2010) found that raising livestock can not only affect the biomass of above-ground vegetation, but also change the community structure of bacteria. Similarly, Lienhard et al. (2013) found that maximum bacterial and fungal diversity occurred with different utilization schemes. Microbial biomass and/or diversity can increase, decrease, or remain the same depending on the type of grassland (Jangid et al., 2008), geographical location (Death and Barquín, 2012), and the grazing system and intensity (Gou et al., 2015). However, some factors were not considered in this study. The vast territory of Inner Mongolia has many reasons for the diversity difference of myxobacteria. In addition to the environmental factors studied in this article, which affect the diversity of myxobacteria, many factors such as heavy metals, salinity, rainfall, and topography may affect the diversity of myxobacteria. Therefore, the factors affecting the structure and diversity of myxobacteria community need to be further studied.

## 5 Conclusion

Arduous isolation work of myxobacterial strains is the premise for research and development of their metabolic capabilities in industry and medicine, and requires a global view of their geographic diversity and distribution, which, however, is lacking. Therefore, the community structure, diversity and the community function of myxobacteria in Inner Mongolia were studied by high-throughput sequencing technology. In a word, there were abundant myxobacteria resources in the soils of Inner Mongolia, and the richness and diversity of samples from the central to eastern regions were significantly higher than those from the western regions. The myxobacteria resources in the whole region included 10 families and 22 genera, among which the dominant genera were *Labilitrix*, *Sandaracinus*, *Archangium*, and *Haliangium*. The distribution, type and nutrient composition of soil samples had an impact on the composition and abundance of the species in soil samples. The changes in myxobacteria community were mainly attributed to the latitude and longitude of the sampling point, as well as pH value, contents of water, available phosphorus, and organic matter in the soil. The results of this study are an important step to understand the effect of soil distribution on myxobacteria community, and also provide basic data for in-depth research on myxobacteria resources. The underlying adaptation mechanisms for pandemic and endemic distribution patterns of myxobacteria under different taxonomic hierarchy still need to be explored.

## Data Availability

The datasets presented in this study can be found in online repositories. The names of the repository/repositories and accession number(s) can be found here: NCBI, under accession number PRJNA1112089 (https://www.ncbi.nlm.nih.gov/bioproject/PRJNA1112089/).
