# Supplementary material for: Community structure and diversity of myxobacteria in soils from Inner Mongolia, China
Source: Front Microbiol. 2025 Jan 22;15:1501573. doi: 10.3389/fmicb.2024.1501573 (PMC11794786; doi:10.3389/fmicb.2024.1501573)
Supplement: Supplementary file 1 [file Data_Sheet_1.docx]

**Supplementary Table 1** The basic information of soil samples

| Group | Soil sample number | Sampling position | Soil type | Soil utilization way | Northern latitude  （°） | East longitude（°） | Altitude（m） |
| --- | --- | --- | --- | --- | --- | --- | --- |
| I | AL-2 | Alxa League | Shruby meadow soil | [Unutilized land](https://dict.youdao.com/w/eng/scattered_plots_of_unutilized_land/#keyfrom=dict.phrase.wordgroup) | 42.03413 | 100.63947 | 939 |
|  | AL-3 | Alxa League | Shruby meadow soil | [Unutilized land](https://dict.youdao.com/w/eng/scattered_plots_of_unutilized_land/#keyfrom=dict.phrase.wordgroup) | 42.03413 | 100.63947 | 939 |
|  | AL-4 | Alxa League | Shruby meadow soil | Farmland | 41.96801 | 100.65245 | 946 |
|  | AL-5 | Alxa League | Shruby meadow soil | Grassland | 41.96846 | 100.65309 | 946 |
|  | AL-1 | Alxa League | Grey brown desert soil | Farmland | 41.94012 | 100.62837 | 951 |
|  | AL-6 | Alxa League | Grey brown desert soil | [Unutilized land](https://dict.youdao.com/w/eng/scattered_plots_of_unutilized_land/#keyfrom=dict.phrase.wordgroup) | 41.98637 | 100.37673 | 950 |
|  | AL-7 | Alxa League | Grey brown desert soil | [Unutilized land](https://dict.youdao.com/w/eng/scattered_plots_of_unutilized_land/#keyfrom=dict.phrase.wordgroup) | 41.98637 | 100.37673 | 950 |
|  | AL-12 | Alxa League | Grey brown desert soil | Farmland | 42.03367 | 101.10879 | 923 |
|  | AL-13 | Alxa League | Grey brown desert soil | Non-cultivated land | 42.03268 | 101.11083 | 924 |
|  | AL-11 | Alxa League | Red willow forest soil | woodland | 42.04222 | 101.05948 | 922 |
|  | AL-14 | Alxa League | Populus euphratica forest soil | woodland | 42.0275 | 101.09863 | 926 |
|  | AL-9 | Alxa League | Saline | [Unutilized land](https://dict.youdao.com/w/eng/scattered_plots_of_unutilized_land/#keyfrom=dict.phrase.wordgroup) | 41.76873 | 100.98833 | 954 |
|  | AL-10 | Alxa League | Saline | [Unutilized land](https://dict.youdao.com/w/eng/scattered_plots_of_unutilized_land/#keyfrom=dict.phrase.wordgroup) | 41.76873 | 100.98833 | 954 |
|  | AL-24 | Alxa League | Gray desert soil | Pastoral area | 38.80277 | 101.34812 | 2380 |
|  | AL-25 | Alxa League | Gray desert soil | Pastoral area | 38.84484 | 101.33427 | 2140 |
|  | AL-26 | Alxa League | Gray desert soil | Pastoral area | 38.87536 | 101.37003 | 1950 |
|  | AL-20 | Alxa League | Gray desert soil | [Unutilized land](https://dict.youdao.com/w/eng/scattered_plots_of_unutilized_land/#keyfrom=dict.phrase.wordgroup) | 38.31715 | 104.77299 | 1310 |
|  | AL-35 | Alxa League | Gray desert soil | [Unutilized land](https://dict.youdao.com/w/eng/scattered_plots_of_unutilized_land/#keyfrom=dict.phrase.wordgroup) | 40.80801 | 103.63207 | 1320 |
|  | AL-36 | Alxa League | Gray desert soil | Farmland | 40.81707 | 103.67254 | 1330 |
|  | AL-37 | Alxa League | Gray desert soil | Pastoral area | 40.5907 | 103.71576 | 1360 |
|  | AL-34 | Alxa League | Gray desert soil | Unutilized land | 40.80801 | 103.63207 | 1320 |
|  | AL-38 | Alxa League | Gray desert soil | Pastoral area | 39.91375 | 104.9097 | 1540 |
|  | AL-21 | Alxa League | Aeolian sandy soil | Farmland | 38.79192 | 105.38564 | 1290 |
|  | AL-22 | Alxa League | Aeolian sandy soil | Unutilized land | 38.79081 | 105.38581 | 1290 |
|  | AL-23 | Alxa League | Aeolian sandy soil | woodland | 38.7904 | 105.46541 | 1330 |
|  | AL-30 | Alxa League | Aeolian sandy soil | Unutilized land | 39.44892 | 101.98486 | 1420 |
|  | AL-31 | Alxa League | Aeolian sandy soil | Unutilized land | 39.44888 | 101.98536 | 1380 |
|  | AL-33 | Alxa League | Aeolian sandy soil | Unutilized land | 39.73175 | 101.16717 | 1260 |
|  | AL-27 | Alxa League | Chisley soil | Pastoral area | 38.86236 | 101.51315 | 1820 |
|  | AL-28 | Alxa League | Chisley soil | Unutilized land | 38.81189 | 101.50943 | 1930 |
|  | AL-29 | Alxa League | Chisley soil | Unutilized land | 38.81234 | 101.51042 | 1920 |
| II | BM-24 | Bayan Nur | Haloxylon forest soil | woodland | 42.07466 | 106.54176 | 906 |
|  | BM-36 | Bayan Nur | Cumulated irrigated soil | Farmland | 40.8901 | 106.98155 | 1032 |
|  | BM-37 | Bayan Nur | Cumulated irrigated soil | Farmland | 40.89053 | 106.98598 | 1029 |
|  | BM-38 | Bayan Nur | Cumulated irrigated soil | Farmland | 40.80632 | 107.30371 | 1032 |
|  | BM-39 | Bayan Nur | Cumulated irrigated soil | Non-cultivated land | 40.80631 | 107.30367 | 1030 |
|  | ERDS-15 | Ordos | Moisture soil | Farmland | 40.45212 | 110.23244 | 1000 |
|  | ERDS-16 | Ordos | Moisture soil | Grassland | 40.45279 | 110.23184 | 1007 |
|  | ERDS-17 | Ordos | Moisture soil | Farmland | 40.47536 | 109.5498 | 1000 |
|  | ERDS-18 | Ordos | Moisture soil | Grassland | 40.47485 | 109.55035 | 999 |
|  | ERDS-23 | Ordos | Brown calcic soil | Farmland | 40.13201 | 107.79682 | 1140 |
|  | ERDS-24 | Ordos | Brown calcic soil | Grassland | 40.13464 | 107.79644 | 1136 |
|  | ERDS-26 | Ordos | Brown calcic soil | Farmland | 39.37997 | 107.82823 | 1349 |
|  | ERDS-27 | Ordos | Brown calcic soil | Grassland | 39.37736 | 107.83038 | 1348 |
|  | ERDS-28 | Ordos | Sierozem | Grassland | 38.09156 | 107.41935 | 1322 |
|  | ERDS-29 | Ordos | Sierozem | Farmland | 38.09319 | 107.41402 | 1318 |
|  | ERDS-30 | Ordos | Sierozem | woodland | 39.22915 | 106.92062 | 1385 |
|  | ERDS-31 | Ordos | Sierozem | Farmland | 38.23106 | 106.91935 | 1389 |
|  | ERDS-32 | Ordos | Sierozem | Grassland | 38.23182 | 106.91688 | 1384 |
| III | WLCB-4 | Ulanqab | Castano-cinnamon soil | Farmland | 40.43701 | 112.48096 | 1273 |
|  | WLCB-5 | Ulanqab | Castano-cinnamon soil | Grassland | 40.43407 | 112.48174 | 1282 |
|  | WLCB-1 | Ulanqab | Chestnut soil | Farmland | 40.67154 | 112.29726 | 1363 |
|  | WLCB-2 | Ulanqab | Chestnut soil | woodland | 40.65619 | 112.29807 | 1456 |
|  | WLCB-3 | Ulanqab | Chestnut soil | Grassland | 40.65883 | 112.31149 | 1402 |
|  | WLCB-13 | Ulanqab | Chestnut soil | Farmland | 40.92613 | 113.28322 | 1293 |
|  | WLCB-14 | Ulanqab | Chestnut soil | Grassland | 40.92557 | 113.28335 | 1297 |
|  | WLCB-15 | Ulanqab | Chestnut soil | Farmland | 40.9147 | 113.25683 | 1291 |
|  | WLCB-16 | Ulanqab | Chestnut soil | Grassland | 40.91437 | 113.25629 | 1288 |
|  | WLCB-17 | Ulanqab | Chestnut soil | Grassland | 41.49436 | 113.18114 | 1411 |
|  | WLCB-18 | Ulanqab | Chestnut soil | Farmland | 41.66604 | 112.83817 | 1380 |
|  | WLCB-19 | Ulanqab | Chestnut soil | Grassland | 41.66779 | 112.82463 | 1374 |
|  | WLCB-20 | Ulanqab | Chestnut soil | Grassland | 41.82188 | 112.31445 | 1544 |
|  | WLCB-21 | Ulanqab | Chestnut soil | Farmland | 41.8218 | 112.31473 | 1543 |
|  | WLCB-28 | Ulanqab | Chestnut soil | Farmland | 41.75189 | 111.27257 | 1515 |
|  | WLCB-29 | Ulanqab | Chestnut soil | Grassland | 41.75272 | 111.27245 | 1516 |
|  | WLCB-6 | Ulanqab | Grey-cinnamon soil | Grassland | 40.37215 | 112.87236 | 1406 |
|  | WLCB-7 | Ulanqab | Grey-cinnamon soil | Farmland | 40.37209 | 112.88101 | 1377 |
|  | WLCB-32 | Ulanqab | Grey-cinnamon soil | Farmland | 41.15978 | 111.98602 | 1749 |
|  | WLCB-33 | Ulanqab | Grey-cinnamon soil | Grassland | 41.15472 | 111.985 | 1745 |
|  | WLCB-34 | Ulanqab | Grey-cinnamon soil | Farmland | 40.99812 | 112.07242 | 1329 |
|  | WLCB-35 | Ulanqab | Grey-cinnamon soil | Grassland | 40.99775 | 112.0719 | 1334 |
|  | XLGL-2 | Xilingol League | Brown calcic soil | Grassland | 42.20923 | 112.81374 | 1255 |
|  | XLGL-6 | Xilingol League | Brown calcic soil | Grassland | 44.02923 | 112.07031 | 1100 |
|  | XLGL-9 | Xilingol League | Brown calcic soil | Grassland | 44.01941 | 113.12218 | 943 |
|  | HHHT-21 | Hohhot | Alluvial soil | Grassland | 40.18507 | 111.79062 | 1111 |
|  | HHHT-22 | Hohhot | Alluvial soil | Farmland | 40.1852 | 111.79013 | 1104 |
| IV | CF-8 | Chifeng | Grey forest soil | Farmland | 43.31591 | 117.44247 | 1143 |
|  | CF-9 | Chifeng | Grey forest soil | Grassland | 43.31602 | 117.44245 | 1143 |
|  | CF-14 | Chifeng | Grey forest soil | Farmland | 43.03136 | 117.64044 | 1140 |
|  | CF-15 | Chifeng | Grey forest soil | Grassland | 43.03125 | 117.64048 | 1135 |
|  | CF-16 | Chifeng | Grey forest soil | woodland | 43.03142 | 117.64052 | 1145 |
|  | CF-11 | Chifeng | Skeletal soil | Grassland | 43.47418 | 117.67885 | 1158 |
|  | CF-12 | Chifeng | Skeletal soil | Farmland | 43.47375 | 117.67928 | 1156 |
|  | CF-13 | Chifeng | Skeletal soil | woodland | 43.47514 | 117.6786 | 1159 |
|  | CF-23 | Chifeng | Skeletal soil | woodland | 42.00689 | 118.06663 | 1040 |
|  | CF-24 | Chifeng | Skeletal soil | Grassland | 42.99688 | 118.06658 | 1040 |
|  | CF-25 | Chifeng | Skeletal soil | Farmland | 42.99658 | 118.06663 | 1038 |
|  | CF-32 | Chifeng | Brunisolic soil | Grassland | 41.70691 | 118.37328 | 931 |
|  | CF-33 | Chifeng | Brunisolic soil | woodland | 41.70715 | 118.37318 | 935 |
|  | CF-34 | Chifeng | Brunisolic soil | Farmland | 41.70706 | 118.37326 | 936 |
|  | CF-35 | Chifeng | Brunisolic soil | Grassland | 41.68018 | 118.29797 | 1049 |
|  | CF-36 | Chifeng | Brunisolic soil | woodland | 41.68027 | 118.29798 | 1046 |
|  | CF-37 | Chifeng | Brunisolic soil | Farmland | 41.68027 | 118.29788 | 1044 |
|  | CF-29 | Chifeng | Cinnamon soil | Grassland | 41.84592 | 118.50916 | 846 |
|  | CF-30 | Chifeng | Cinnamon soil | Farmland | 41.84558 | 118.50947 | 838 |
|  | CF-31 | Chifeng | Cinnamon soil | Farmland | 41.84557 | 118.50945 | 839 |
|  | CF-38 | Chifeng | Cinnamon soil | Grassland | 41.68333 | 119.0511 | 631 |
|  | CF-39 | Chifeng | Cinnamon soil | woodland | 41.68365 | 119.05111 | 636 |
|  | CF-40 | Chifeng | Cinnamon soil | Farmland | 41.68369 | 119.0521 | 639 |
|  | CF-44 | Chifeng | Cinnamon soil | Grassland | 42.31027 | 120.63245 | 480 |
|  | CF-45 | Chifeng | Cinnamon soil | woodland | 42.31189 | 120.63287 | 456 |
|  | CF-46 | Chifeng | Cinnamon soil | Farmland | 42.31208 | 120.63418 | 456 |
|  | CF-47 | Chifeng | Cinnamon soil | Farmland | 42.2443 | 120.7835 | 410 |
|  | CF-48 | Chifeng | Cinnamon soil | Unutilized land | 43.24415 | 120.78334 | 408 |
| V | XAM-3 | Hinggan League | Chernozem | Grassland | 45.99017 | 120.52045 | 680 |
|  | XAM-4 | Hinggan League | Chernozem | Farmland | 45.99873 | 120.52033 | 680 |
|  | XAM-5 | Hinggan League | Bog soil | Farmland | 45.8252 | 120.30438 | 753 |
|  | XAM-6 | Hinggan League | Bog soil | Grassland | 45.28522 | 120.30132 | 753 |
|  | XAM-8 | Hinggan League | Bog soil | Farmland | 44.99355 | 121.33006 | 359 |
|  | XAM-9 | Hinggan League | Bog soil | Unutilized land | 44.99382 | 121.33259 | 366 |
|  | XAM-15 | Hinggan League | Bog soil | Farmland | 44.7362 | 122.02948 | 189 |
|  | XAM-16 | Hinggan League | Bog soil | Grassland | 44.73612 | 122.02977 | 187 |
|  | XAM-1 | Hinggan League | Dark brown soil | Grassland | 45.95856 | 120.45342 | 702 |
|  | XAM-2 | Hinggan League | Dark brown soil | Farmland | 45.95977 | 120.45737 | 699 |
|  | XAM-12 | Hinggan League | Dark brown soil | woodland | 45.05905 | 121.48634 | 270 |
|  | HLBE-9 | Hulun Buir | Dark brown soil | Farmland | 47.34584 | 121.85731 | 564 |
|  | HLBE-10 | Hulun Buir | Dark brown soil | woodland | 47.34632 | 121.85786 | 561 |
|  | XAM-17 | Hinggan League | Meadow soil | Farmland | 44.44501 | 122.00753 | 178 |
|  | XAM-18 | Hinggan League | Meadow soil | Grassland | 44.44495 | 122.00638 | 179 |
|  | XAM-19 | Hinggan League | Meadow soil | Farmland | 44.73137 | 121.82294 | 188 |
|  | XAM-20 | Hinggan League | Meadow soil | Grassland | 44.73289 | 121.82976 | 190 |
|  | XAM-27 | Hinggan League | Meadow soil | Farmland | 46.28761 | 122.75039 | 244 |
|  | XAM-28 | Hinggan League | Meadow soil | Grassland | 46.28787 | 122.75035 | 244 |
|  | XAM-36 | Hinggan League | Meadow soil | Grassland | 44.7631 | 120.32143 | 770 |
|  | XAM-25 | Hinggan League | Meadow chernozem | Farmland | 45.56039 | 122.48299 | 328 |
|  | XAM-26 | Hinggan League | Meadow chernozem | Grassland | 45.56007 | 122.483 | 327 |
|  | XAM-30 | Hinggan League | Phaeozem | Farmland | 46.74012 | 121.92457 | 401 |
|  | XAM-31 | Hinggan League | Phaeozem | Grassland | 46.74068 | 121.92136 | 402 |
|  | XAM-32 | Hinggan League | Phaeozem | Farmland | 46.50279 | 121.60027 | 497 |
|  | XAM-33 | Hinggan League | Phaeozem | Grassland | 46.50234 | 121.60079 | 497 |
|  | HLBE-17 | Hulun Buir | Brown coniferous forest soil | woodland | 51.86821 | 121.86701 | 708 |
|  | HLBE-19 | Hulun Buir | Brown coniferous forest soil | woodland | 51.51406 | 121.71329 | 759 |
|  | HLBE-20 | Hulun Buir | Brown coniferous forest soil | woodland | 51.51408 | 121.71332 | 762 |

AL: Alxa League. BM: Bayan Nur. ERDS: Ordos. WLCB: Ulanqab. XLGL: Xilingol League. HHHT: Hohhot. CF: Chifeng. XAM: Hinggan League. HLBE: Hulun Buir.

**Supplementary Table 2** The Parameters of soil samples

| Soil sample number | The content of water（%） | pH value | The content of organic matter（g/kg） | The content of available potassium  （mg/kg） | The content of available phosphorus（mg/kg） | The content of hydrolyzed nitrogen  （mg/kg） |
| --- | --- | --- | --- | --- | --- | --- |
| AL-2 | 0.89±0.010 | 7.50±0.030 | 9.979±0.614 | 87.136±2.084 | 14.028± 1.968 | 19.600±3.704 |
| AL-3 | 0.62±0.006 | 7.43±0.065 | 7.094±0.424 | 65.469±6.402 | 12.044± 0.320 | 30.566±5.064 |
| AL-4 | 6.00±0.050 | 7.31±0.100 | 24.268±0.769 | 34.790±1.839 | 27.442±0.083 | 23.566±2.829 |
| AL-5 | 1.09±0.011 | 7.60±0.050 | 33.398±1.555 | 91.457±3.529 | 9.150±0.445 | 17.233±0.839 |
| AL-1 | 8.29±0.049 | 7.69±0.015 | 14.289±0.567 | 136.241±2.985 | 33.011±6.121 | 32.900±0.989 |
| AL-6 | 1.88±0.006 | 6.75±0.089 | 28.520±0.271 | 235.630±4.815 | 9.204±0.489 | 65.133±2.397 |
| AL-7 | 0.22±0.006 | 6.59±0.045 | 8.146±0.057 | 41.426±1.582 | 9.732±1.447 | 13.650±1.485 |
| AL-12 | 9.75±0.057 | 7.72±0.021 | 14.493±0.102 | 67.630±5.847 | 21.708±0.301 | 61.603±2.428 |
| AL-13 | 1.49±0.011 | 7.99±0.070 | 14.120±0.579 | 185.870±1.296 | 9.750±0.395 | 45.033±3.453 |
| AL-11 | 0.98±0.037 | 8.04±0.072 | 10.861±0.327 | 240.506±7.273 | 18.942±1.863 | 26.600±3.704 |
| AL-14 | 2.82±0.016 | 8.37±0.025 | 25.286±0.734 | 297.914±15.659 | 29.062± 0.192 | 126.933±7.081 |
| AL-9 | 1.94±0.028 | 7.35±0.065 | 15.375±0.358 | 251.123±2.081 | 13.190±1.480 | 37.333±2.139 |
| AL-10 | 0.68±0.050 | 7.95±0.050 | 16.461±0.817 | 292.420±6.840 | 11.825±1.455 | 35.933±2.914 |
| AL-24 | 11.30±0.003 | 7.47±0.031 | 29.291±0.424 | 147.784±7.154 | 9.332±0.289 | 64.400±3.704 |
| AL-25 | 4.17±0.002 | 7.36±0.055 | 20.602±0.367 | 280.444±2.422 | 12.135±0.032 | 36.633±1.457 |
| AL-26 | 5.96±0.001 | 7.93±0.040 | 23.657±0.367 | 175.685±7.649 | 16.758± 0.570 | 42.466±0.808 |
| AL-20 | 9.55±0.074 | 8.15±0.076 | 14.595±0.305 | 62.815±4.271 | 16.648±0.751 | 27.066±2.139 |
| AL-35 | 0.28±0.001 | 8.01±0.040 | 7.399±0.358 | 38.123±1.317 | 5.182±0.055 | 8.400±1.400 |
| AL-36 | 10.49±0.002 | 7.68±0.030 | 15.070±0.156 | 123.988±9.223 | 7.057±0.477 | 36.866±2.914 |
| AL-37 | 0.87±0.004 | 7.87±0.020 | 8.248±0.424 | 113.432±3.889 | 5.182± 0.055 | 1.350±0.071 |
| AL-34 | 0.47±0.004 | 7.97±0.067 | 6.211±0.385 | 96.858±9.042 | 7.057±0.477 | 13.566±1.589 |
| AL-38 | 1.84±0.001 | 7.94±0.060 | 13.475±0.269 | 71.966±2.881 | 7.057± 0.477 | 6.666±0.577 |
| AL-21 | 8.03±0.046 | 7.18±0.065 | 23.385±1.171 | 106.395±5.635 | 13.809±0.902 | 54.100±6.495 |
| AL-22 | 0.73±0.158 | 7.57±0.057 | 7.908±0.512 | 125.191±10.670 | 12.863±0.158 | 6.950±0.071 |
| AL-23 | 0.40±0.010 | 7.70±0.046 | 13.135±0.962 | 130.994±5.975 | 8.804±1.266 | 14.050±0.071 |
| AL-30 | 0.20±0.000 | 7.32±0.053 | 3.157±0.327 | 41.272±1.297 | 2.961±0.280 | 4.900±0.990 |
| AL-31 | 1.97±0.003 | 7.67±0.035 | 11.947±0.367 | 42.506±3.354 | 5.182±0.055 | 6.533±0.808 |
| AL-33 | 0.38±0.000 | 7.82±0.040 | 6.653±0.103 | 98.247±7.158 | 5.182±0.055 | 3.500±0.990 |
| AL-27 | 4.62±0.009 | 7.64±0.078 | 22.537±0.833 | 191.580±2.769 | 11.461± 0.447 | 40.133±2.914 |
| AL-28 | 3.39±0.000 | 7.83±0.035 | 17.208±0.358 | 28.340±0.899 | 8.458±0.189 | 38.850±0.495 |
| AL-29 | 2.66±0.002 | 7.97±0.031 | 9.300±0.367 | 61.951±3.601 | 5.819± 0.441 | 14.050±0.071 |
| BM-24 | 0.55±0.03 | 8.21±0.02 | 6.82±0.16 | 109.72±0.35 | 77.11±0.23 | 40.96±2.09 |
| BM-36 | 15.95±0.03 | 7.88±0.01 | 15.92±1.2 | 113.50±2.64 | 53.00±1.35 | 68.07±2.15 |
| BM-37 | 13.8±0.22 | 8.15±0.02 | 19.52±0.51 | 152.87±1.29 | 110.69±0.41 | 24.24±2.15 |
| BM-38 | 11.15±0.06 | 7.79±0.01 | 16.19±0.78 | 93.96±2.13 | 45.55±0.17 | 25.16±1.39 |
| BM-39 | 5.33±0.03 | 8.18±0.02 | 18.77±0.73 | 143.30±0.55 | 21.45±1.74 | 26.10±2.13 |
| ERDS-15 | 13.49±0.34 | 7.47±0.07 | 10.79±0.20 | 72.58±4.57 | 18.18±0.60 | 38.90±1.38 |
| ERDS-16 | 20.23±0.13 | 7.52±0.03 | 12.72±0.54 | 99.64±2.82 | 12.57±0.98 | 32.80±0.81 |
| ERDS-17 | 19.77±0.24 | 7.39±0.06 | 20.43±0.92 | 115.50±3.02 | 11.44±0.51 | 37.47±0.36 |
| ERDS-18 | 25.89±0.96 | 8.00±0.04 | 22.47±0.66 | 148.39±1.01 | 6.97±0.22 | 33.70±1.40 |
| ERDS-23 | 7.22±0.13 | 7.80±0.05 | 12.32±0.68 | 106.63±2.50 | 7.53±0.37 | 27.95±1.38 |
| ERDS-24 | 5.01±0.36 | 8.63±0.09 | 12.90±0.41 | 55.28±0.31 | 4.38±0.26 | 14.4±1.76 |
| ERDS-26 | 5.77±0.53 | 7.41±0.12 | 14.12±0.41 | 89.82±1.45 | 7.58±0.41 | 40.28±2.42 |
| ERDS-27 | 5.39±0.08 | 7.26±0.10 | 12.42±0.36 | 99.29±0.86 | 5.36±0.64 | 29.37±1.37 |
| ERDS-28 | 2.28±0.03 | 7.63±0.05 | 7.60±0.16 | 30.52±1.75 | 4.82±0.26 | 19.09±1.60 |
| ERDS-29 | 2.99±0.10 | 7.56±0.03 | 7.84±0.21 | 79.47±1.20 | 5.31±0.11 | 29.78±0.84 |
| ERDS-30 | 1.05±0.10 | 7.80±0.05 | 7.84±0.16 | 47.62±1.28 | 4.53±0.45 | 14.66±1.86 |
| ERDS-31 | 3.94±0.24 | 7.75±0.02 | 9.64±0.42 | 114.20±0.93 | 8.39±0.38 | 54.10±0.04 |
| ERDS-32 | 0.37±0.11 | 7.59±0.07 | 8.15±0.26 | 94.12±1.01 | 5.25±0.84 | 15.14±1.07 |
| WLCB-4 | 4.95±0.92 | 7.25±0.01 | 10.33±0.29 | 10.82±0.12 | 42.29±0.17 | 36.63±0.49 |
| WLCB-5 | 4.32±0.13 | 7.48±0.01 | 8.10±0.28 | 11.55±0.12 | 18.91±0.37 | 74.91±0.11 |
| WLCB-1 | 4.60±0.22 | 7.65±0.01 | 6.85±0.12 | 11.66±0.24 | 18.20±0.14 | 53.79±0.41 |
| WLCB-2 | 5.34±0.15 | 6.84±0.05 | 13.63±0.12 | 8.17±0.12 | 43.43±0.14 | 18.81±0.24 |
| WLCB-3 | 3.73±0.23 | 7.16±0.02 | 10.88±0.02 | 10.75±0.33 | 35.26±0.36 | 188.53±0.35 |
| WLCB-13 | 8.67±0.79 | 7.47±0.01 | 17.25±0.16 | 14.00±0.12 | 65.81±0.53 | 65.05±0.45 |
| WLCB-14 | 7.63±0.41 | 7.39±0.04 | 18.07±0.24 | 10.38±0.02 | 62.57±0.41 | 82.30±0.36 |
| WLCB-15 | 20.05±0.42 | 7.13±0.01 | 24.21±0.09 | 18.51±0.12 | 58.19±0.14 | 68.26±0.24 |
| WLCB-16 | 10.24±0.37 | 6.94±0.03 | 18.80±0.12 | 17.58±0.33 | 336.24±0.12 | 247.28±0.33 |
| WLCB-17 | 5.20±0.02 | 8.21±0.04 | 14.93±0.17 | 15.48±0.33 | 97.83±0.61 | 248.41±0.37 |
| WLCB-18 | 13.28±0.38 | 7.06±0.02 | 19.84±0.04 | 15.66±0.02 | 116.26±0.32 | 37.67±0.46 |
| WLCB-19 | 7.68±0.02 | 7.18±0.02 | 34.57±0.12 | 16.37±0.29 | 95.73±0.09 | 230.67±0.94 |
| WLCB-20 | 5.99±1.20 | 7.05±0.03 | 15.36±0.72 | 16.55±0.53 | 33.84±0.36 | 41.08±0.32 |
| WLCB-21 | 9.53±0.22 | 7.23±0.01 | 14.15±0.24 | 14.56±0.28 | 41.85±0.09 | 57.97±0.74 |
| WLCB-28 | 4.11±1.07 | 6.86±0.02 | 14.41±0.16 | 15.04±0.29 | 50.27±0.26 | 84.35±0.38 |
| WLCB-29 | 4.27±1.04 | 7.27±0.05 | 18.02±0.32 | 10.09±0.12 | 39.91±0.77 | 41.13±0.12 |
| WLCB-6 | 13.26±0.67 | 7.76±0.01 | 11.51±0.04 | 9.43±0.12 | 36.94±0.02 | 15.96±0.66 |
| WLCB-7 | 14.63±0.96 | 7.43±0.04 | 15.59±0.26 | 18.60±0.36 | 73.57±0.04 | 38.75±0.28 |
| WLCB-32 | 9.53±0.29 | 6.95±0.02 | 32.27±0.04 | 19.64±0.38 | 104.57±0.17 | 62.25±0.35 |
| WLCB-33 | 15.57±1.48 | 7.23±0.08 | 26.88±0.33 | 19.79±0.24 | 50.26±0.49 | 147.92±0.82 |
| WLCB-34 | 20.52±1.29 | 7.42±0.02 | 32.93±0.17 | 42.79±0.41 | 77.29±0.65 | 157.09±0.92 |
| WLCB-35 | 8.72±0.70 | 7.07±0.02 | 42.74±0.33 | 16.77±0.03 | 112.94±0.38 | 140.83±0.35 |
| XLGL-2 | 1.08±0.12 | 7.06±0.46 | 8.56±0.12 | 9.56±0.17 | 30.66±0.49 | 195.55±0.69 |
| XLGL-6 | 2.88±0.02 | 6.85±0.02 | 9.01±0.24 | 8.56±0.09 | 15.67±0.02 | 66.23±0.07 |
| XLGL-9 | 5.13±0.27 | 6.87±0.02 | 11.13±0.24 | 12.19±0.14 | 3.72±0.28 | 109.34±0.41 |
| HHHT-21 | 3.77±0.12 | 7.97±0.05 | 21.82±1.45 | 15.09±0.41 | 75.13±0.07 | 182.15±0.32 |
| HHHT-22 | 3.38±0.43 | 7.92±0.05 | 16.79±1.62 | 16.73±0.65 | 63.00±0.21 | 76.14±1.34 |
| CF-8 | 10.74±0.57 | 7.32±0.16 | 8.12±0.87 | 2.80±0.75 | 1.26±0.10 | 61.81±0.69 |
| CF-9 | 10.07±0.67 | 6.56±0.20 | 12.22±0.93 | 23.73±0.85 | 2.38±0.37 | 83.00±0.43 |
| CF-14 | 8.52±0.48 | 6.92±0.12 | 7.43±0.33 | 66.39±0.17 | 3.00±0.03 | 48.60±0.42 |
| CF-15 | 9.23±0.32 | 7.02±0.15 | 13.07±0.52 | 60.58±0.39 | 0.59±0.02 | 55.77±0.67 |
| CF-16 | 8.28±0.24 | 6.94±0.01 | 14.14±0.64 | 194.62±0.55 | 0.98±0.26 | 69.76±0.65 |
| CF-11 | 8.05±0.35 | 6.82±0.10 | 10.28±1.46 | 25.21±0.95 | 1.43±0.29 | 76.23±0.09 |
| CF-12 | 7.76±0.23 | 6.81±0.01 | 11.94±0.82 | 73.14±2.25 | 3.50±0.02 | 69.43±0.22 |
| CF-13 | 6.55±0.17 | 6.25±0.11 | 16.21±0.94 | 196.66±1.92 | 1.75±0.17 | 62.09±0.30 |
| CF-23 | 8.70±0.30 | 7.02±0.04 | 5.83±1.55 | 78.00±1.69 | 3.70±0.27 | 55.97±0.94 |
| CF-24 | 13.31±0.13 | 7.09±0.04 | 10.25±1.01 | 25.14±0.53 | 1.16±0.09 | 56.18±1.24 |
| CF-25 | 13.19±0.75 | 6.67±0.73 | 12.60±0.63 | 115.98±0.63 | 4.99±0.51 | 55.78±0.80 |
| CF-32 | 7.01±0.37 | 6.51±0.05 | 4.89±0.61 | 12.76±0.63 | 4.89±1.42 | 62.37±0.10 |
| CF-33 | 9.47±0.18 | 6.75±0.15 | 15.67±0.36 | 263.98±0.76 | 20.39±1.00 | 112.04±1.04 |
| CF-34 | 11.92±0.26 | 5.64±0.21 | 17.93±0.20 | 171.03±0.18 | 21.36±1.52 | 132.76±0.64 |
| CF-35 | 12.36±0.29 | 5.36±0.15 | 32.04±0.82 | 156.41±1.27 | 6.94±0.97 | 188.93±0.89 |
| CF-36 | 23.23±0.67 | 6.27±0.09 | 46.02±0.80 | 0.08±0.07 | 6.15±0.30 | 209.59±0.42 |
| CF-37 | 24.69±0.09 | 5.83±0.06 | 34.10±0.36 | 3.80±0.34 | 4.93±0.30 | 174.34±0.05 |
| CF-29 | 7.01±0.41 | 7.03±0.25 | 11.137±0.22 | 60.94±0.35 | 2.38±0.14 | 90.48±0.26 |
| CF-30 | 7.93±0.24 | 6.99±0.01 | 8.12±0.68 | 61.15±3.19 | 3.98±0.15 | 83.92±0.87 |
| CF-31 | 9.17±0.37 | 6.89±0.36 | 7.30±0.85 | 14.21±0.76 | 9.09±1.00 | 76.76±0.64 |
| CF-38 | 12.68±0.19 | 6.52±0.08 | 34.61±0.52 | 34.10±0.54 | 0.82±0.09 | 90.62±0.45 |
| CF-39 | 12.48±0.52 | 6.55±0.11 | 19.53±0.76 | 5.89±0.32 | 1.37±0.12 | 98.18±1.24 |
| CF-40 | 9.95±0.18 | 6.54±0.00 | 21.53±0.59 | 20.99±2.04 | 3.98±0.18 | 69.30±0.00 |
| CF-44 | 2.04±0.06 | 6.72±0.10 | 13.79±0.61 | 101.90±0.96 | 6.09±0.13 | 83.76±0.52 |
| CF-45 | 3.73±0.12 | 6.50±0.16 | 8.90±0.46 | 31.17±0.34 | 0.73±0.20 | 55.69±0.54 |
| CF-46 | 3.63±0.21 | 6.54±0.09 | 9.72±0.46 | 29.54±0.24 | 5.45±0.20 | 62.16±0.20 |
| CF-47 | 4.11±0.06 | 6.73±0.02 | 9.72±0.22 | 48.09±1.25 | 4.74±0.89 | 83.55±0.35 |
| CF-48 | 3.36±0.06 | 7.05±0.01 | 11.16±0.44 | 55.28±0.53 | 1.19±0.44 | 48.48±0.25 |
| XAM-3 | 17.36±0.32 | 6.27±0.07 | 36.92±0.38 | 21.62±1.63 | 11.20±0.58 | 108.36±1.46 |
| XAM-4 | 15.27±1.64 | 5.78±0.05 | 40.59±0.52 | 20.81±1.41 | 12.19±0.33 | 61.60±0.81 |
| XAM-5 | 18.91±0.42 | 5.71±0.03 | 41.88±0.50 | 22.05±0.73 | 15.64±0.10 | 52.48±0.40 |
| XAM-6 | 26.26±0.18 | 5.51±0.10 | 52.97±0.09 | 55.06±1.53 | 11.95±0.58 | 94.40±0.85 |
| XAM-8 | 31.65±1.25 | 7.39±0.02 | 54.67±0.58 | 25.67±1.66 | 5.06±0.26 | 32.94±0.70 |
| XAM-9 | 25.53±0.28 | 6.15±0,04 | 55.64±0.32 | 34.02±1.24 | 4.95±0.42 | 28.52±0.85 |
| XAM-15 | 12.80±0.32 | 8.43±0.08 | 12.10±0.90 | 4.81±1.74 | 3.09±0.29 | 17.40±0.40 |
| XAM-16 | 10.81±0.82 | 8.15±0.11 | 10.69±0.63 | 3.57±1.50 | 2.37±0.06 | 17.26±0.00 |
| XAM-1 | 15.33±0.41 | 5.82±0.02 | 48.37±0.53 | 41.08±1.69 | 11.86±0.13 | 70.86±0.62 |
| XAM-2 | 15.27±4.28 | 5.44±0.01 | 35.77±0.78 | 37.60±1.73 | 14.53±0.08 | 89.84±0.17 |
| XAM-12 | 10.12±1.11 | 6.01±0.01 | 10.06±0.47 | 16.36±0.68 | 2.95±0.81 | 17.54±0.81 |
| HLBE-9 | 13.00±0.82 | 4.68±0.03 | 31.38±0.04 | 73.74±0.05 | 1.88±0.02 | 325.17±0.15 |
| HLBE-10 | 20.55±0.43 | 4.14±0.11 | 32.76±0.02 | 90.05±0.11 | 1.32±0.02 | 209.85±0.23 |
| XAM-17 | 9.70±0.24 | 8.23±0.04 | 7.68±0.14 | 1.84±1.02 | 2.67±0.06 | 17.12±0.40 |
| XAM-18 | 9.65±0.18 | 7.95±0.09 | 8.21±0.38 | 6.13±1.60 | 3.72±0.24 | 24.10±0.70 |
| XAM-19 | 12.48±0.19 | 8.81±0.03 | 12.48±0.41 | 8.57±0.76 | 2.78±0.19 | 28.24±0.40 |
| XAM-20 | 11.86±0.06 | 8.34±0.06 | 10.85±0.41 | 4.07±1.02 | 2.90±0.27 | 26.24±0.46 |
| XAM-27 | 12.30±0.72 | 7.05±0.06 | 40.56±0.13 | 4.82±1.69 | 0.84±0.11 | 20.33±0.88 |
| XAM-28 | 8.05±0.18 | 7.26±0.03 | 41.53±0.62 | 3.77±1.25 | 0.19±0.06 | 35.21±0.90 |
| XAM-36 | 12.42±0.70 | 5.06±0.04 | 54.54±0.57 | 31.54±1.67 | 11.33±0.56 | 27.90±1.02 |
| XAM-25 | 10.93±0.86 | 6.55±0.04 | 42.72±1.51 | 14.64±1.45 | 0.90±0.12 | 83.84±0.81 |
| XAM-26 | 9.17±0.21 | 4.72±0.14 | 44.60±0.30 | 10.17±1.67 | 0.46±0.11 | 37.08±1.07 |
| XAM-30 | 12.80±0.63 | 5.08±0.07 | 24.29±0.81 | 20.71±0.82 | 13.42±0.15 | 50.22±0.89 |
| XAM-31 | 17.11±1.64 | 4.87±0.05 | 39.18±0.10 | 21.24±1.43 | 5.68±0.22 | 31.33±0.57 |
| XAM-32 | 16.08±0.85 | 4.66±0.04 | 41.38±0.41 | 8.33±0.20 | 3.70±0.13 | 42.14±1.03 |
| XAM-33 | 12.30±0.62 | 5.00±0.05 | 49.40±0.16 | 16.15±1.71 | 3.45±0.25 | 30.70±0.80 |
| HLBE-17 | 15.36±1.77 | 3.81±0.02 | 34.61±0.12 | 95.74±0.12 | 1.89±0.02 | 153.61±0.12 |
| HLBE-19 | 7.82±0.18 | 5.11±0.13 | 28.21±0.01 | 104.06±0.11 | 4.66±0.09 | 256.45±0.43 |
| HLBE-20 | 7.47±0.18 | 4.95±0.26 | 15.83±0.01 | 90.09±0.05 | 2.74±0.05 | 109.60±0.52 |

**Supplementary Table 3** Grading standards of soil pH and nutrients in the second national soil survey in China

| The nutrient indicators of soil | I | II | III | IV | V | VI |
| --- | --- | --- | --- | --- | --- | --- |
| Water content  （%） |  | wet | Suitable | Light drought | Moderate drought | Heavy drought |
|  |  | ＞20 | 16~20 | 12~16 | 6~12 | ﹤6 |
| pH value | Strong alkaline | Slightly alkaline | Neutral | Slightly acidic | Strong acidic | Extremely acidic |
|  | ＞8.5 | 7.5~8.5 | 6.5~7.5 | 5.5~6.5 | 4.5~5.5 | ﹤4.5 |
| Content of hydrolyzed nitrogen（mg/kg） | Extremely high | High | Moderate | Low | Very low | Extremely low |
|  | ＞150 | 120~150 | 90~120 | 60~90 | 30~60 | ﹤30 |
| Content of available potassium（mg/kg） | Extremely high | High | Moderate | Low | Very low | Extremely low |
|  | ＞200 | 150~200 | 100~150 | 50~100 | 30~50 | ﹤30 |
| Content of organic matter  （g/kg） | Extremely high | High | Moderate | Low | Very low | Extremely low |
|  | ＞40 | 30~40 | 20~30 | 10~20 | 6~10 | ﹤6 |
| Content of available phosphorus（mg/kg） | Extremely high | High | Moderate | Low | Very low | Extremely low |
|  | ＞40 | 20~40 | 10~20 | 5~10 | 3~5 | ﹤3 |

**
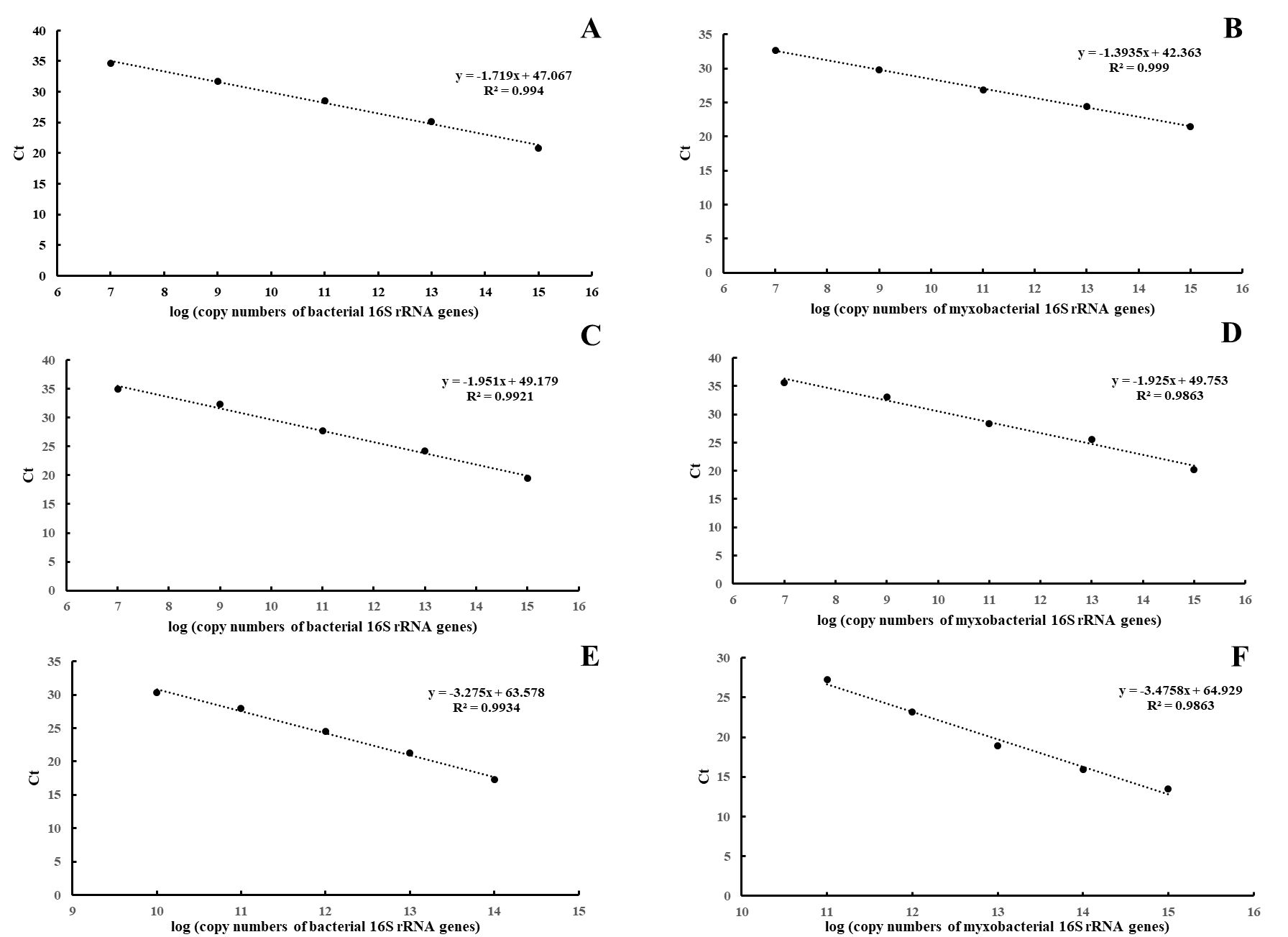
**

**Supplementary** **Figure 1** The standard curves. (A) Standard curve for amplification using *Sorangium cellulosum* S-C12 as template and 1369F/1541R as primers; (B) Standard curve for amplification using *Sorangium cellulosum* S-C12 as template and W5/802R as primers; (C) Standard curve for amplification using *Nannocystis exedens* DSM14640 as template and 1369F/1541R as primers; (D) Standard curve for amplification using *Nannocystis exedens* DSM14640 as template and W5/802R as primers; (E) Standard curve for amplification using *Myxococcus xanthus* BM30 as template and 1369F/1541R as primers; (F) Standard curve for amplification using *Myxococcus xanthus* BM30 as template and W5/802R as primers;.


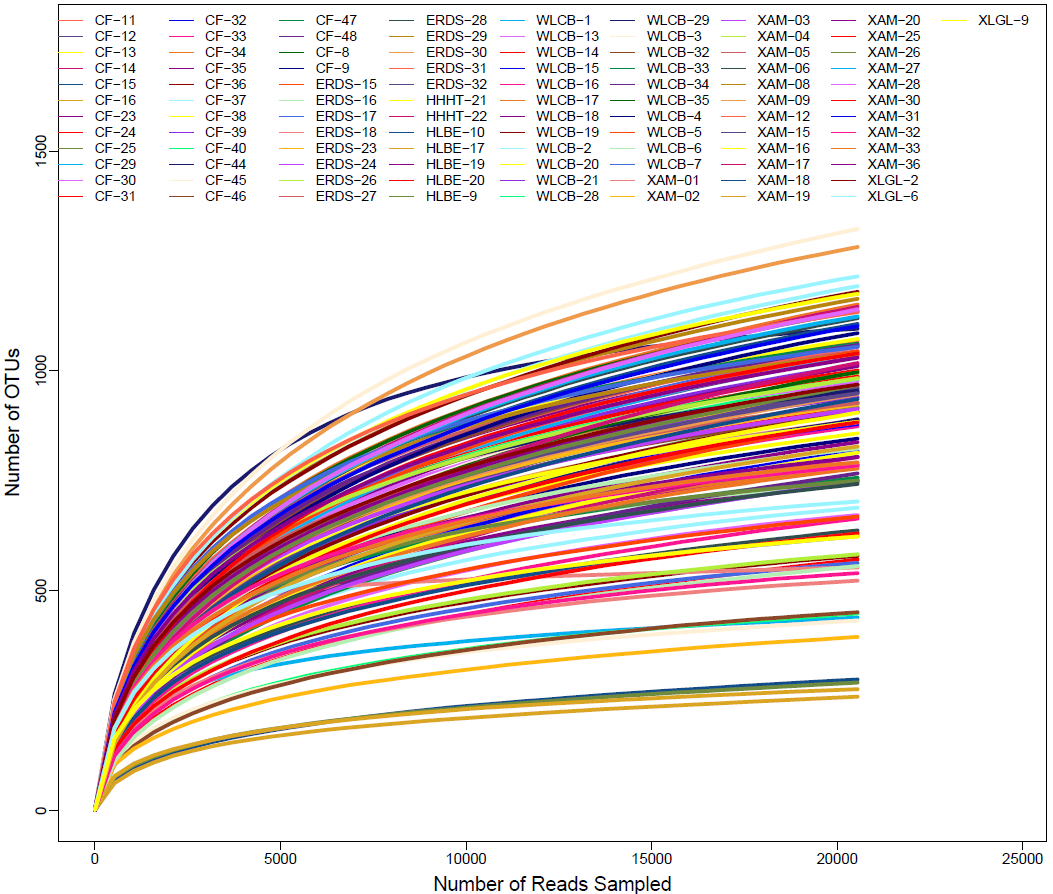


**Supplementary** **Figure 2** The rarefaction curves. The abscissa represents the number of sequences randomly selected from the sample, and the ordinate represents the number of OTUs to which the extracted sequences belong. Each curve in the figure represents a sample, which is marked with a different color. The 16S rRNA gene sequences of myxobacteria are available at the NCBI GenBank under accession number PRJNA1112089.

**Supplementary Table 4** Relative abundance of species at genus level

| Genus | Relative Abundance（%） | | | | |
| --- | --- | --- | --- | --- | --- |
|  | Group I | Group II | Group III | Group IV | Group V |
| Unassigned | 59.45 | 59.02 | 65.28 | 64.61 | 63.30 |
| *Archangium* | 10.05 | 5.88 | 4.64 | 3.27 | 3.09 |
| *Sandaracinus* | 8.25 | 8.89 | 5.38 | 5.60 | 4.17 |
| *Haliangium* | 5.02 | 7.81 | 4.45 | 2.00 | 2.89 |
| *Labilithrix* | 4.8 | 4.34 | 5.60 | 7.36 | 11.04 |
| *Kofleria* | 1.87 | 2.32 | 2.49 | 2.44 | 2.50 |
| *Cystobacter* | 1.83 | 1.54 | 0.71 | 0.51 | 0.64 |
| *Nannocystis* | 1.08 | 0.79 | 0.76 | 0.73 | 0.40 |
| *Polyangium* | 1.02 | 1.62 | 1.92 | 2.18 | 2.09 |
| *Sorangium* | 0.97 | 1.23 | 1.82 | 2.10 | 1.68 |
| *Myxococcus* | 0.88 | 1.04 | 0.77 | 0.42 | 0.35 |
| *Minicystis* | 0.85 | 0.55 | 2.24 | 1.78 | 3.46 |
| *Chondromyces* | 0.85 | 1.46 | 0.69 | 2.37 | 0.60 |
| *Byssovorax* | 0.69 | 0.77 | 1.14 | 3.21 | 2.44 |
| *Aggregicoccus* | 0.63 | 0.32 | 0.39 | 0.41 | 0.38 |
| *Enhygromyxa* | 0.50 | 1.00 | 0.61 | 0.31 | 0.11 |
| *Anaeromyxobacter* | 0.49 | 0.57 | 0.47 | 0.29 | 0.25 |
| *Jahnella* | 0.32 | 0.15 | 0.03 | 0.02 | 0.01 |
| *Phaselicystis* | 0.29 | 0.56 | 0.23 | 0.24 | 0.42 |
| *Stigmatella* | 0.07 | 0.06 | 0.13 | 0.10 | 0.11 |
| *Pseudenhygromyxa* | 0.06 | 0.01 | 0.01 | 0.01 | 0.004 |
| *Vulgatibacter* | 0.04 | 0.08 | 0.19 | 0.05 | 0.08 |
| *Hyalangium* | 0.01 | 0.002 | 0.04 | 0.01 | 0.01 |
